# Metal transporter Slc30a1 controls pharyngeal neural crest differentiation via the zinc‐Snai2‐Jag1 cascade

**DOI:** 10.1002/mco2.91

**Published:** 2021-09-27

**Authors:** Zhidan Xia, Xinying Bi, Sisi Yang, Xiu Yang, Zijun Song, Jiayu Wei, Pengfei Xu, Lothar Rink, Junxia Min, Fudi Wang

**Affiliations:** ^1^ The First Affiliated Hospital School of Public Health Institute of Translational Medicine Institute of Genetics Zhejiang University School of Medicine Hangzhou China; ^2^ The First Affiliated Hospital Hengyang Medical School University of South China Hengyang China; ^3^ Faculty of Medicine Institute of Immunology RWTH Aachen University Aachen Germany

**Keywords:** neural crest, pharyngeal arch, Slc30a1, zinc homeostasis, zebrafish

## Abstract

The pharyngeal arch (PA) is a neural crest (NC)‐derived organ that is transiently developed during embryogenesis and is required for the subsequent development of various tissues. However, the role of zinc during PA differentiation from NC progenitor cells is unknown. Here, we found that the metal transporters Slc30a1a and Slc30a1b mediate zinc homeostasis during PA differentiation. Slc30a1‐deficient zebrafish develop zinc accumulation in NC cells, with increased expression of stemness markers and PA dorsal genes, and SMART‐seq analyses revealed that the genes *snai2* and *jag1b* may serve as downstream targets. Furthermore, functional studies showed that knocking down either *snai2* or *jag1b* rescues PA development in Slc30a1‐deficient zebrafish. Notably, we identified the double zinc‐finger domain in the transcription factor Snai2 as a zinc‐responsive element that regulates *jag1b* expression. Our findings indicate that the Slc30a1/zinc‐*snai2*‐*jag1b* axis is an essential regulatory network controlling PA differentiation, shedding new light on the function of zinc homeostasis in maintaining NC cell stemness and multipotency in vertebrates.

## Graphical abstract

A novel role of Slc30a1/zinc in neural crest differentiation is reported. In animals lacking Slc30a1, zinc accumulates in neural crest progenitors, thereby upregulating the expression of *jag1b* via the double zinc‐finger in the Snai2 protein. The findings shed light on the function of zinc homeostasis in the regulation of neural crest multipotency in vivo, providing valuable targets in diagnosing and treating congenital birth defects.

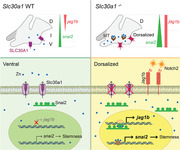

## 1 INTRODUCTION

In vertebrates, the pharyngeal arch (PA) is an essential structure formed during early development, giving rise to a variety of tissues in the face and neck region.^1^ Pharyngeal cartilage is an essential supporting structure derived from the pharyngeal neural crest.^1^, ^2^ During this process, neural crest cells (NCCs) migrate out of neural folds via an epithelial‐to‐mesenchymal transition, which results in their transformation into multipotent progenitor cells.^3^, ^4^ These progenitor cells then continue their migration to the craniofacial region, where they condense and proliferate, forming chondrogenic progenitors,^5^, ^6^ which divide further into subpopulations along the dorsoventral (DV) axis, with cells in each subpopulation expressing specific molecular signals.^7^, ^8^ In particular, the endothelin‐1 (Edn1), bone morphogenetic protein (BMP), and Jagged‐Notch pathways are the most thoroughly characterized signaling pathways expressed along the DV region of the PA, and their proper interactions ensure the normal differentiation of pharyngeal cartilage.^9^, ^10^, ^11^, ^12^ However, the factors that drive the development of NCCs in forming pharyngeal chondrocytes are not fully characterized.

Zinc, an essential trace element, is an integral component of many proteins, including metalloenzymes and zinc‐finger (ZF)‐containing transcription factors, and the regulatory role that zinc plays in development and metabolism has been thoroughly explored.^13^, ^14^, ^15^ Recently, a growing body of in vitro evidence supports the notion that zinc plays an important role in regulating stem cell fate; however, the results arising from different studies are inconsistent. For example, some studies found that zinc can help stem cells maintain certain properties, including self‐renewal and pluripotency,^16^, ^17^ whereas other studies found that zinc can suppress certain stem cell properties.^18^, ^19^ Nevertheless, how zinc—and zinc‐regulated pathways—drives stem cell fate in vivo remains an open question.

Slc30a and Slc39a are two major families of zinc transporters, playing important roles in maintaining zinc homeostasis.^20^, ^21^, ^22^ For example, Slc30a1 knockout mice are embryonic lethal, suggesting that this protein is essential during early development.^23^ In addition, a mutation in the *s* *lc30a1* orthologue *s* *lc30a1a* causes delayed embryonic development in zebrafish.^24^ In *Drosophila*, Slc30a1 is expressed in the basolateral membrane of enterocytes, mediating the uptake of dietary zinc.^25^ Finally, in cultured cells SLC30A1 is expressed at the cell membrane, where it functions as a zinc exporter.^26^, ^27^ To date, however, the precise biological function of Slc30a1 in vertebrates is poorly understood.

In humans, defects in the NC and PA account for a wide range of birth defects.^1^ Despite clinical studies that suggest a strong association between an imbalance in maternal zinc homeostasis and the risk of birth defects in the developing infant,^28^, ^29^, ^30^, ^31^ whether zinc homeostasis plays a role in determining the fate of NCCs remains unclear. Here, we investigated the role of Slc30a1 and zinc in mediating the development of pharyngeal NCCs. In zebrafish embryos, we found that knocking out both *slc30a1a* and *slc30a1b* causes a failure of PA differentiation, as well as increased stemness and decreased differentiation signals in NC progenitor cells. Interestingly, we also found that zinc accumulates in these arrested progenitor cells. Finally, we identified *snai2* and *jag1b* as two downstream targets of Slc30a1.

Our results indicate that Slc30a1‐mediated zinc homeostasis plays an essential role in controlling NC stemness state and PA differentiation by targeting *snai2* and *jag1b*, revealing a novel regulatory pathway, as well as providing insight into potential new targets for diagnosing and treating congenital craniofacial defects.

## 2 RESULTS

### 2.1 *slc30a1a/slc30a1b* double‐knockout zebrafish develop severe PA malformations

**FIGURE 1:**
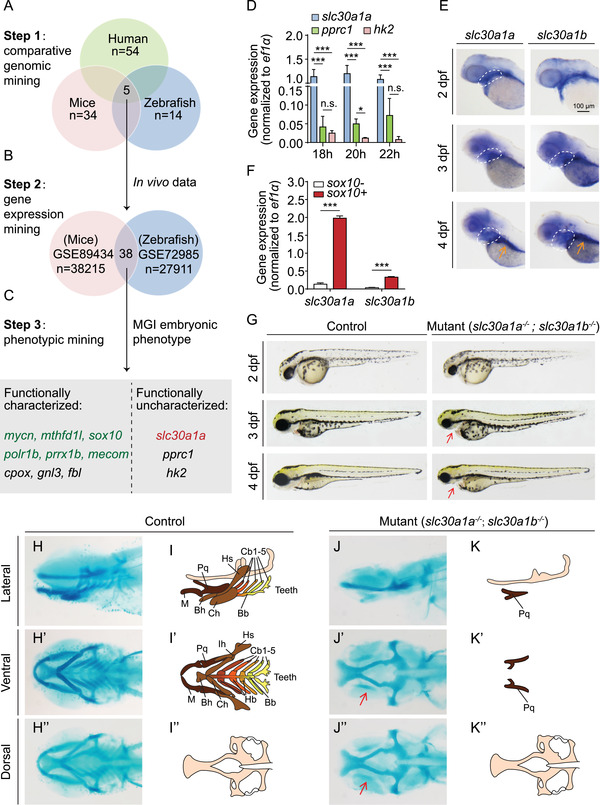
Generation and characterization of *slc30a1a*/*slc30a1b* double‐knockout zebrafish. (A) Strategy used to perform comparative genomic mining for neural crest (NC)‐related studies using human, mouse, and zebrafish data, resulting in five overlapping studies. (B) Two of the five studies identified in (A) were selected based on in vivo experiments and data involving multiple time points, revealing a total of 38 overlapping genes in these two databases. These genes were then ranked based on their expression levels. (C) Embryonic phenotype mining revealed 12 candidate genes. Nine of these genes have been characterized in vivo, while the other three genes have not been characterized at the functional level. The six genes in green are known to play a role in NC development (*mycn*, *mthfd1l*, *sox10*, *polr1b*, and *mecom*) and/or pharyngeal arch (PA) patterning (*sox10* and *prrx1b*). (D) Real‐time quantitative polymerase chain reaction (RT‐qPCR) analysis of the indicated genes at 18–22 hpf. (E) in situ hybridization of wild‐type zebrafish embryos using antisense *slc30a1a* and *slc30a1b* probes. The white dashed outlines indicate the pharyngeal region, and the gut is indicated with an orange arrow in the 4 dpf images. (F) Real‐time qPCR analysis of *slc30a1a* and *slc30a1b* mRNA in *sox10* ^+^ and *sox10^−^* cells (*n* = 3 sets of 3 × 10^4^ cells/group). ***p < 0.001. (G) Representative images of *slc30a1a*/*slc30a1b* double‐knockout mutants and control embryos. The red arrows in the mutant embryos indicate a smaller head and loss of the lower jaw. (H–K) Representative images of head cartilage in control and mutant zebrafish embryos stained with Alcian blue (H and J) and the corresponding diagrams (I and K). Residual palatoquadrate cartilage in the mutant embryos is indicated with red arrows. Abbreviations for cartilage: M, Meckel's; Pq, palatoquadrate; Bh, basihyal; Ch, ceratohyal; Hs, hyosymplectic; Bb, basibranchial; Cb, ceratobranchial; Hb, hypobranchial; Ih, interhyal

To identify potential candidate genes that may play a role in NC development, we performed a series of knowledge‐based data mining experiments. We started with comparative genomic mining based on gene expression omnibus (GEO) datasets related to the NC in humans, mice, and zebrafish. Five studies containing RNA‐seq databases (Figure 1A and Figure S1A) were analyzed further. Among these five GEO datasets, two in vivo datasets (GSE72985 and GSE89434) containing multiple time points were analyzed further based on gene expression mining.^32^, ^33^ Because the DV patterning of NCCs in the PA region begins at approximately 24 hour post‐fertilization (24 hpf) in zebrafish and at embryonic day 9 (E9) in mice,^8^ we performed gene set enrichment analysis by extracting the data at two critical developmental time points in zebrafish (20 and 36 hpf) and mice (E8.5 and 10.5), reflecting the temporal progression of DV patterning (Figure 1B and Figure S1A). As shown in Figure 1B, of the 38 overlapping genes between mice and zebrafish, 12 genes were ranked for potential candidate genes (Figure 1C and Figure S1A,B). Importantly, six of these 12 genes (shown in green in Figure 1C) were previously reported to function in the NC, with *sox10* and *mycn* serving as markers for multipotent NCCs.^11^, ^34^, ^35^, ^36^, ^37^, ^38^ Moreover, the detailed phenotypes of zebrafish carrying mutant or morphant versions of three genes (*gnl3*, *fbl*, and *cpox*) have been reported.^39^, ^40^, ^41^, ^42^ Among the three uncharacterized genes, we found that the expression of *slc30a1a* was the highest during early development (Figure 1D). In addition, *slc30a1a* was particularly interesting as a candidate gene, as it encodes a putative zinc transporter, and zinc has been linked previously to cell stemness.^16^, ^17^ We, therefore, focused on studying the function of *slc30a1a*—and its paralog, *slc30a1b*—in NC development in zebrafish.

Using semi‐quantitative polymerase chain reaction (PCR), we found that *slc30a1a* is expressed at stable levels through 7 days post‐fertilization (7 dpf), whereas the expression of its paralog *slc30a1b* decreases from 1 dpf through 7 dpf (Figure S1C). Using in situ hybridization, we found that both *slc30a1a* and *slc30a1b* are expressed robustly in the pharyngeal region at 2–4 dpf and in the gut at 4 dpf (Figure 1E), suggesting that they might have redundant functions. Next, we used the transgenic zebrafish line *Tg(sox10:kikGR)*, in which NCCs are specifically labeled with a fluorescent tag,^43^ and measured the expression levels of *slc30a1a* and *slc30a1b* in fluorescent (*sox10* ^+^) and non‐fluorescent (*sox10* ^‒^) cells in zebrafish embryo heads. We found that the expression levels of both the *slc30a1a* and *slc30a1b* genes were significantly higher in *sox10* ^+^ cells compared to *sox10* ^–^ cells (Figure 1F).

To study the function of Slc30a1 proteins in zebrafish development, we then generated mutant zebrafish lines in which either the *slc30a1a* or *slc30a1b* gene was knocked out using CRISPR/Cas9 (Figure S2A). We found no clear phenotype in either homozygous *slc30a1a* or homozygous *slc30a1b* knockout embryos during early developmental stages. Interestingly, however, the homozygous *slc30a1a* knockout zebrafish died due to an unknown cause before reaching sexual maturity; in contrast, homozygous *slc30a1b* knockout animals grew normally to adulthood. Considering the possibility of redundancy between these two genes during early development, we then crossed heterozygous *slc30a1a* knockout animals with homozygous *slc30a1b* knockout animals to generate a double heterozygous *slc30a1a^+/−^*/*slc30a1b^+/−^* knockout line (Figure S2A). We then intercrossed the double heterozygous adults and analyzed the resulting offspring (Figure S2B). We found that a small number of embryos had a phenotype including microcephalia with small eyes. We then further performed genotyping and Alcian blue staining in order to examine the putative correlation between genotype and phenotype. We found that all of the embryos with microcephalia and small eyes were double homozygous *slc30a1^−/−^*/*slc30a1b^−/−^*, while the embryos with a normal phenotype had a variety of other genotypes (Figure S2B). To increase the ratio of double homozygous mutants for further analysis, we intercrossed *slc30a1a^+/−^*/*slc30a1b^−/−^* zebrafish (Figure S2C). We then determined the number of embryos with microcephalia and small eyes and matched this phenotype with the double homozygous knockout offspring (*slc30a1a* ^−/−^/*slc30a1b* ^−/−^) identified by genotyping. As expected, based on Mendelian inheritance, one‐quarter of the offspring were double homozygous knockout (Figure S2D).

These double homozygous knockout mutants (hereafter referred to simply as “mutants”) developed microcephalia at 2 dpf, and showed a clear reduced lower jaw during later development (Figure 1G). All the mutants have a dramatic viscerocranium deficiency (Figure 1H–K), in which nearly all of the seven paired PAs are lost, with only a small amount of palatoquadrate cartilage remaining (Figure 1J–J', K–K'), despite an intact neurocranium (Figure 1J'', K''). In addition, the mutants showed similar body length to their controls (Figure S3A), however, they did not develop PAs even in later stages (Figure S3B), suggesting the lack of PAs is not due to a developmental delay in mutants. As the mutants did not develop a functional mouth, they could not survive to adulthood.

To eliminate the possibility of an off‐target lesion introduced by CRISPR, we screened two separate editing alleles for each slc30a1 paralog (Figure S3C–F). We found that all mutant alleles had sufficient knockout efficiency, and both editing strategies yielded the same PA phenotype (Figure S3G). In addition, in situ hybridization of *slc30a1a* confirmed knockout in the mutants (Figure S3H), and co‐injecting plasmids expressing wild‐type *slc30a1a* and *slc30a1b* partially rescued the PA dysplasia phenotype (Figure S3I,J). Taken together, these data indicate that both *slc30a1a* and *slc30a1b* play a critical role in PA development.

### 2.2 *slc30a1a*/*slc30a1b* double‐knockout zebrafish embryos have impaired PA differentiation

**FIGURE 2:**
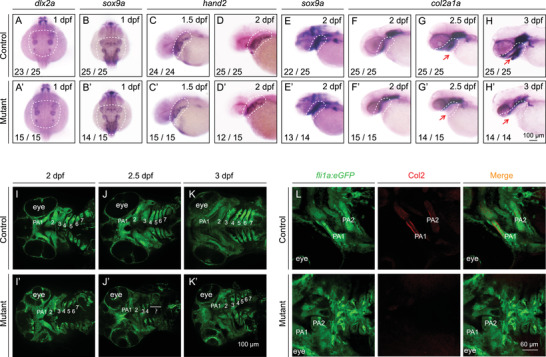
Pharyngeal arch development is impaired in *slc30a1a*/*slc30a1b* double‐knockout embryos. (A–H) The expression pattern of the indicated genes was determined in control and mutant embryos using whole‐mount in situ hybridization. Shown are dorsal views from anterior to the top (A and B) and lateral views from anterior to the left (C–H). The dashed white regions indicate the location of neural crest (NC) progenitors (A–D) and pharyngeal archs (PAs) (E–H). (I–K) Pharyngeal cartilage in control and mutant *Tg(fli1a:eGFP)* embryos at the indicated ages. (L) Col2 immunostaining (red) showing chondrocyte precursors in the PA of control and mutant *Tg(fli1a:eGFP)* embryos

To determine the stage at which PA development is disrupted in double‐knockout embryos, we analyzed the expression patterns of several marker genes—including *foxd3* and *sox10* for NC specification,^44^ *dlx2a* and *hand2* for NC migration,^45^, ^46^ *sox9a* for NC migration and chondrogenic condensation,^47^ and *col2a1a* for chondrocyte differentiation^5^, ^48^ —by performing in situ hybridization at various time points. Although the expression patterns of markers for NCC specification (Figure S3K), migration, and condensation were similar between mutants and controls (Figure 2A–E’), *col2a1a* expression was significantly lower in the lower jaw in the mutants compared to controls (Figure 2F–H’), suggesting reduced differentiation of chondrogenic progenitors.

Next, we used confocal microscopy to visualize detailed structures during PA development. At 2 dpf, the PAs were arranged regularly along the anteroposterior (AP) axis in both the control and mutant embryos, and some PAs had not separated from each other (Figure 2I–I’). However, we observed a striking morphological change in the PAs of mutant embryos at 2.5 dpf, the stage in which chondrogenic progenitors begin to differentiate.^8^ In control embryos, we found that all seven paired PAs separated along the AP axis and extended along the DV axis (Figure 2J). In contrast, in the mutant embryos, the PAs separated along the AP axis but failed to extend fully along the DV axis (Figure 2J’). Indeed, PA pairs 4–7 were barely visible in 2.5 dpf mutant embryos (Figure 2J’). At 3 dpf, all seven PA pairs were fully developed in control embryos, whereas only a fraction of the PA1 was visible in the mutants (Figure 2K–K’). Using Col2 immunostaining to mark chondrogenic progenitors, we found that Col2 expression began in PA pairs 1 and 2 at 56 hpf in controls, whereas this marker was barely detectable in age‐matched mutants (Figure 2L), indicating impaired chondrogenic differentiation in mutant embryos. Based on these findings, we conclude that Slc30a1 is required for PA differentiation.

### 2.3 PA hypogenesis in s*lc30a1a*/s*lc30a1b* double‐knockout mutants is not due to significant changes in either cell proliferation or cell survival

**FIGURE 3:**
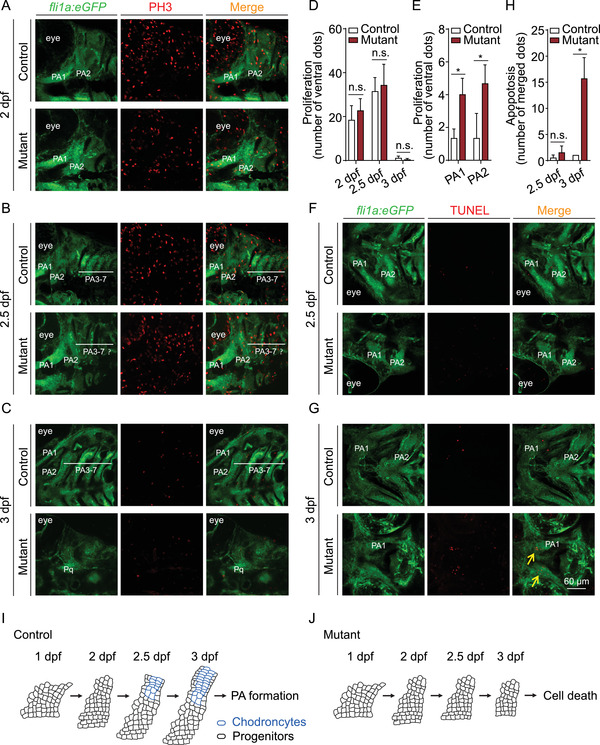
Summary of cell death and cell proliferation in the PA of control and *slc30a1a*/*slc30a1b* double‐knockout mutant embryos. (A–C) PH3 immunostaining (red) was performed in order to detect cell proliferation in the pharyngeal arch (PA) of control and mutant *Tg(fli1a:eGFP)* embryos at the indicated ages. (D) Summary of PH3‐positive cells in the PA of control and mutant embryos at the indicated ages; n.s., not significant. (E) Summary of PH3‐positive cells in the ventral region of PA1 and PA2 in control and mutant embryos at 2 dpf. *p < 0.05. (F and G) A TUNEL assay (red) was used to detect apoptotic cells in the PA area of control and mutant *Tg(fli1a:eGFP)* embryos at the indicated ages. (H) Summary of TUNEL‐positive cells in the PA area of control and mutant embryos at the indicated ages. (I and J) Schematic drawing depicting PA development in control (I) and mutant (J) embryos. Chondrocytes that differentiate from progenitor cells around 2.5 dpf in controls are shown in blue; in embryos, these cells fail to differentiate and do not survive beyond 2.5 dpf

To examine in further detail whether the impaired differentiation observed in mutant PAs is due to decreased proliferation and/or increased apoptosis, we performed immunostaining for the cell proliferation marker PH3 and a TUNEL assay to measure apoptosis. We found that PH3 immunostaining was similar between mutant PAs and control PAs at both 2 dpf (Figure 3A) and 2.5 dpf (Figure 3B), although it is important to note that this proliferation marker was concentrated in the ventral PAs of mutant embryos at 2 dpf (Figure 3A,E). In addition, we observed only weak PH3 immunostaining in the PAs of both control and mutant embryos at 3 dpf (Figure 3C,D), suggesting that PA extension was beginning to slow by this stage of development.

TUNEL staining also revealed no significant differences between control and mutant embryos at 2.5 dpf (Figure 3F). In contrast, robust apoptosis was measured in the mutant embryos—but not in controls—at 3 dpf (Figure 3G,H), suggesting that undifferentiated chondrogenic progenitors cannot survive at this later stage. In addition, the PA development was not recovered in the condition of *p53* knockout, or with ferroptosis or necrosis inhibition in mutants (Figure S4A–C).

These findings suggest that the impaired PA differentiation in mutant embryos is likely, not due to a change in the survival or proliferation of progenitor cells. However, defects in subsequent cell survival may contribute to impaired PA development at a later stage. As illustrated schematically in Figure 3I,J, our results indicate that mutant NCCs lose their chondrogenic differentiation capacity at 2.5 dpf.

### 2.4 SMART‐seq analysis reveals an increased stemness signature in pharyngeal NCCs in *slc30a1a*/*slc30a1b* double‐knockout embryos

**FIGURE 4:**
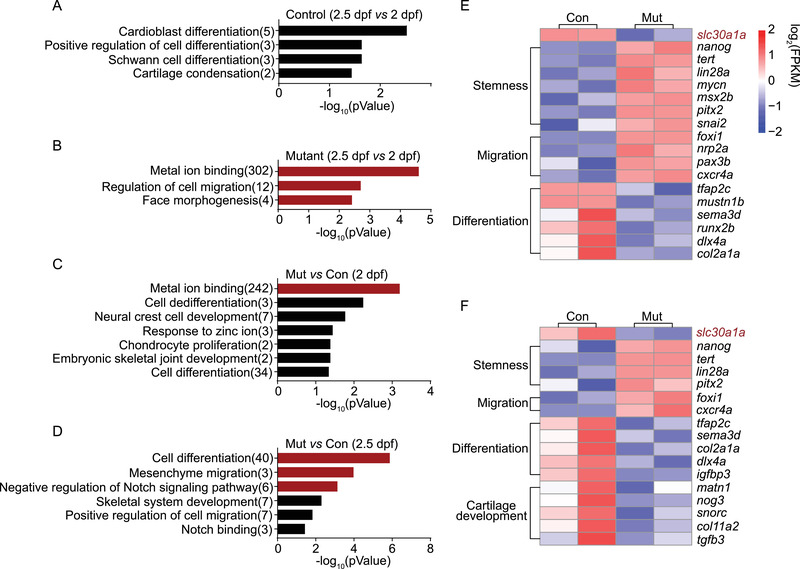
SMART‐seq data analysis of neural crest development and zinc concentration. (A) Summary of significantly enriched pathways (*p *< 0.05) enriched Gene Ontology (GO) terms in control embryos at 2.5 dpf versus control embryos at 2 dpf. In panels A–D, black and red bars indicate *p *< 0.05 and *p *< 0.005, respectively. (B) Summary of the most highly enriched GO terms in mutant embryos at 2.5 dpf versus mutant embryos at 2 dpf. (C and D) Summary of significantly enriched GO terms in mutant embryos versus control embryos at 2 dpf (C) and 2.5 dpf (D). (E and F) Heatmaps showing the expression of the indicated marker genes in control and mutant embryos at 2 dpf (E) and 2.5 dpf (F)

To investigate the molecular mechanisms that underlie PA differentiation, we performed SMART‐seq analysis using *sox10* ^+^ cells isolated from mutant and control embryos at both 2 and 2.5 dpf. We then compared the results obtained at 2.5 dpf with the corresponding results obtained at 2 dpf using Gene Ontology enrichment analysis. We found that in control embryos, NC‐derived tissue differentiation was significantly enriched (Figure 4A and Table S1). In contrast, the most enriched pathways in the mutant embryos were pathways that involve metal ion binding, the regulation of cell migration, and face morphogenesis (Figure 4B and Table S1).

We then compared the mutant and control results at 2 and 2.5 dpf (Figure 4C,D and Table S2). At 2 dpf, the most enriched pathways in the mutant embryos were pathways that involve metal ion binding, cell de‐differentiation, NCC development, response to zinc ions, and chondrocyte proliferation (Figure 4C and Table S2). At 2.5 dpf, the three most enriched pathways were pathways that involve cell differentiation, mesenchyme migration, and negative regulation of the Notch signaling pathway (Figure 4D and Table S2). The pathway involving mesenchymal NC migration was shown previously to play a role in regulating cell pluripotency and self‐renewal.^49^ Moreover, the Notch signaling pathway regulates PA differentiation.^11^ Therefore, our data suggest that the stemness and/or differentiation state of NC progenitors differ between mutant and control embryos.

Next, we measured the gene expression levels in mutant and control embryos at 2 dpf (Figure 4E) and 2.5 dpf (Figure 4F). Our analyses revealed that genes involved in NC stemness and migration are significantly upregulated in mutant embryos at both time points, while genes involved in chondrocyte differentiation and cartilage development are significantly downregulated in mutant embryos at both time points. Consistent with their phenotype, the abnormal development of chondrocytes in mutant embryos may be attributed to the arrest of NC progenitors and the subsequent inhibition of PA differentiation.

### 2.5 Zinc accumulates in pharyngeal NCCs in mutant embryos

**FIGURE 5:**
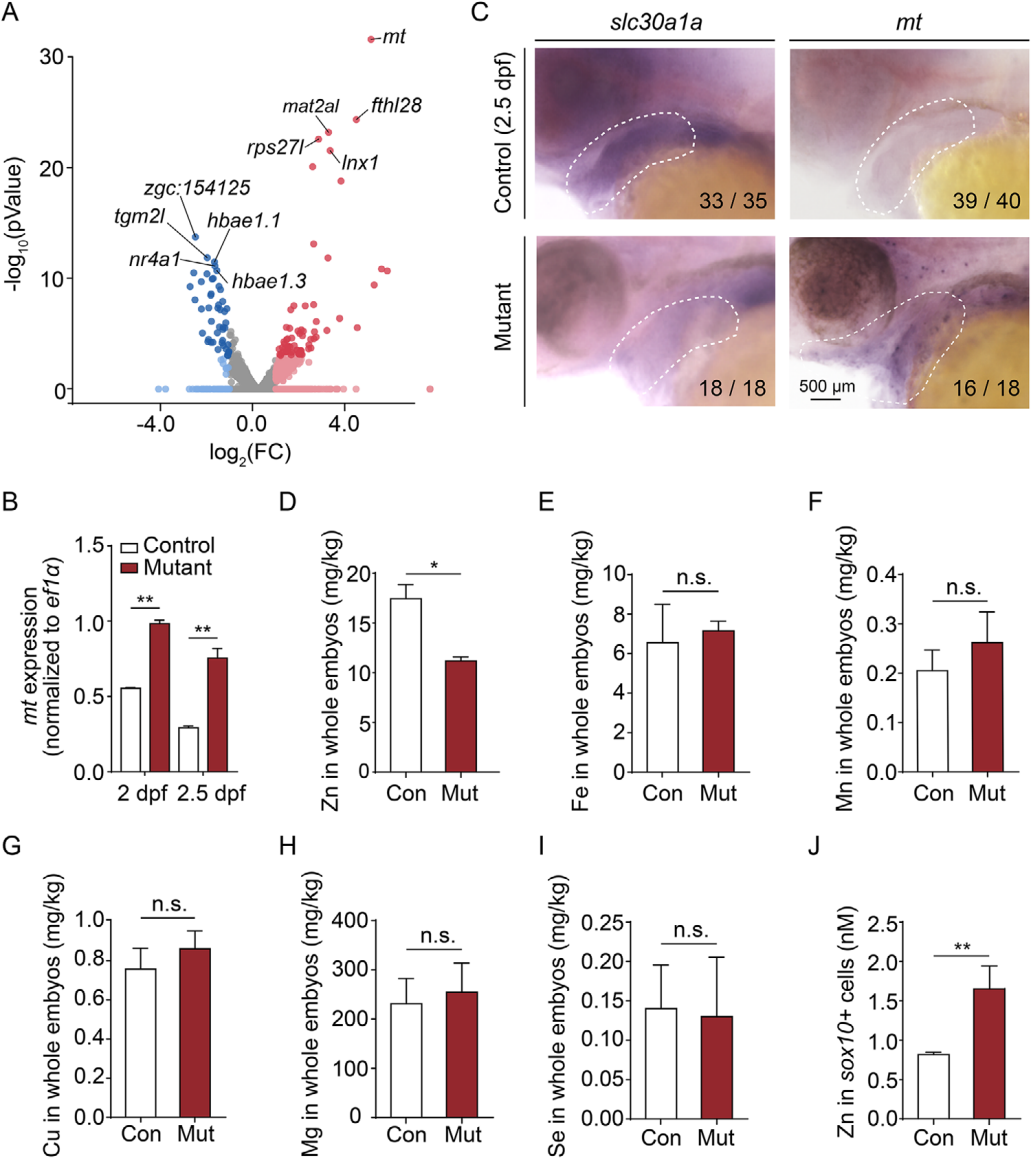
Zinc levels are increased in the pharyngeal arch (PA) of mutant embryos. (A) Volcano plot showing the relative expression of metal ion‐binding genes from Gene Ontology (GO):0046872. Blue and red dots indicate significantly (*p *< 0.05) downregulated and upregulated genes, respectively, and the *mt* gene is indicated. Gray dots indicate genes that were neither upregulated nor downregulated (*p *> 0.05). (B) Summary of *mt* mRNA measured in *sox10* ^+^ cells obtained from control and mutant embryos at 2 dpf and 2.5 dpf. (C) Whole‐mount in situ hybridization of the indicated genes in control and mutant embryos at 2.5 dpf. (D–I) Summary of Zn (D), Fe (E), Mn (F), Cu (G), Mg (H), and Se (I) concentration measured in control and mutant embryos using inductively coupled plasma mass spectrometry (ICP‐MS) (*n* = 2 sets of 100 embryos/group). (J) Summary of Zn concentration measured in *sox10* ^+^ cells isolated from control and mutant embryos at 2 dpf using a fluorescent zinc indicator (*n* = 3 sets of 30,000 cells/group). *p < 0.05, **p < 0.01, n.s., not significant

SLC30A1 is a putative transporter of divalent metal cations. We, therefore, analyzed the cluster of metal ion‐binding genes that our SMART‐seq analysis revealed is enriched in mutant embryos. Interestingly, we found that the *mt* gene (which encodes the metal‐binding protein metallothionein) was the most upregulated gene in the mutant embryos compared to controls (Figure 5A). Consistent with this finding, qPCR analysis confirmed that the expression of *mt* is significantly increased in mutant *sox10* ^+^ cells at both 2 and 2.5 dpf (Figure 5B). Using in situ hybridization, we also found that *mt* mRNA levels were increased in the ventral PA of mutant embryos at 2.5 dpf (Figure 5C).

Given that the Mt protein plays an important role in zinc storage,^50^ we then measured the concentration of various ions in whole embryos using inductively coupled plasma mass spectrometry (ICP‐MS). We found that among the ions analyzed, zinc was the only ion significantly decreased in mutant embryos compared to controls (Figure 5D–I), suggesting that zinc is the principal ion transported by Slc30a1a and/or Slc30a1b in zebrafish. In addition, we found that the cell‐permeable zinc chelator TPEN caused 100% mortality in mutant embryos, but had no effect on control embryos (Figure S4D), suggesting that mutant embryos are more susceptible to zinc deficiency. We also used a fluorescent zinc indicator to measure intracellular zinc levels in *sox10* ^+^ cells isolated from mutant and control embryos. Interestingly, we found significantly higher zinc levels in mutant cells compared to control cells (Figure 5J), consistent with intracellular zinc accumulation. Taken together, these data indicate that zinc, which is transported by Slc30a1 proteins, accumulates primarily in the ventral PA and leads to abnormal PA development in mutant embryos.

### 2.6 Slc30a1a and Slc30a1b regulate PA differentiation via the Jagged‐Notch signaling pathway

**FIGURE 6:**
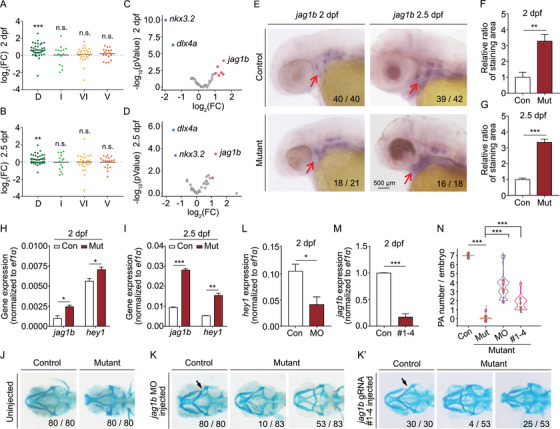
The upregulated Jagged‐Notch signaling inhibits PA development in mutants. (A and B) The domain‐specific difference in gene expression in mutants relative to controls measured along the dorsal/ventral (DV) axis at 2 dpf (A) and 2.5 dpf (B). D, dorsal; I, intermediate; VI, ventral‐intermediate; V, ventral. **p < 0.01 and ***p < 0.001. (C and D) Volcano plots summarizing the difference in expression of marker genes in mutants relative to controls measured along with the anterior/posterior (AP) and DV axes at 2 dpf (C) and 2.5 dpf (D). Blue and red dots indicate significantly (*p *< 0.05) downregulated and upregulated genes, respectively. Gray dots indicate genes that were neither upregulated nor downregulated (*p *> 0.05). The *dlx4a*, *jag1b*, and *nkx3.2* genes are indicated. (E–G) Whole‐mount in situ hybridization of *jag1b* in control and mutant embryos at 2 and 2.5 dpf (E), and summary of the staining area at 2 dpf (F) and 2.5 dpf (G), normalized to the respective controls. (H and I) Summary of *jag1b* and *hey1* mRNA measured in *sox10* ^+^ cells isolated from control and mutant embryos at 2 dpf (H) and 2.5 dpf (I). (J and K) Representative images of head cartilage staining in control and mutant embryos (J) and embryos injected with the *jag1b* MO (K) or a set of four CRISPR/Cas9 ribonucleoprotein complexes (K’). Examples of two distinct mutant phenotypes, with the corresponding frequencies, are shown in K and K’. The arrows in K and K’ indicate the characteristic “kink” in the control embryos. (L) Summary of *hey1* mRNA measured in *sox10* ^+^ cells isolated from control embryos and control embryos injected with the *jag1b* MO. (M) Summary of *jag1b* mRNA measured in control embryos and control embryos injected with the *jag1b* CRISPR/Cas9 ribonucleoprotein complexes. (N) Violin plot summarizing the number of PA pairs/embryos in control embryos, mutant embryos, and mutant embryos injected with either the *jag1b* MO or the *jag1b* CRISPR/Cas9 ribonucleoprotein complexes

The differentiation of pharyngeal chondrocytes is dependent upon spatially defined gene expression along the DV axis.^32^ We, therefore, examined the expression of domain signature genes along the DV axis. We found that dorsal genes are significantly upregulated in the mutant embryos compared to age‐matched controls at both 2 and 2.5 dpf (Figure 6A,B and Table S3), indicating that the PA develops a “dorsalized” pattern in mutant embryos.

Next, we analyzed canonical regulatory signaling pathways related to PA development along the AP and DV axes using volcano plots (Table S4).^8^, ^51^, ^52^ We found that the *dlx4a* and *nkx3.2* genes were downregulated, while the *jag1b* gene was upregulated, in mutant embryos compared to controls (Figure 6C,D). These findings are consistent with previous reports that the *nkx3.2* gene is a marker for joint development and that the expression of both *nkx3.2* and *dlx4a* is suppressed by *jag1b* during PA differentiation.^7^, ^12^ As an internal control, we found that the expression of ventral marker *hand2* ^8^ was similar between mutant and control embryos (Figure S4E).

To examine whether the decreased expression of *dlx4a* and/or the increased expression of *jag1b* contributes to the impaired PA development in mutant zebrafish embryos, we analyzed the expression pattern of these two genes using in situ hybridization. Consistent with our SMART‐seq data, we found that *dlx4a* was significantly downregulated in the PA (Figure S4F). Interestingly, we also found a more diffuse pattern of *jag1b* expression that included the ventral PA in mutant embryos at both 2 and 2.5 dpf (Figure 6E). Moreover, quantitative analyses revealed that *jag1b* expression was significantly higher in the PA region in mutant embryos compared to controls at both time points (Figure 6F,G). Consistent with these results, quantitative PCR analysis confirmed that *dlx4a* is downregulated (Figure S4G) and *jag1b* is upregulated in mutant embryos compared to age‐matched controls (Figure 6H,I). However, overexpressing *dlx4a* in mutant embryos failed to rescue PA formation (Figure S4H–J). To determine whether the increased *jag1b* expression in the mutant embryos causes impaired PA differentiation, we used a morpholino (MO) to block the translation of *jag1b* mRNA,^53^ as well as a set of four CRISPR/Cas9 ribonucleoprotein complexes to decrease transcription of the *jag1b* gene.^54^

Because the *jag1b* MO has been shown to cause a phenotype similar to *jag1b* mutants,^12^ we first examined the skeletal phenotype in our morphants to confirm the efficacy of the MO. We found that *jag1b* morphants develop a phenotype that includes the characteristic kink (Figure 6J–K’) and reduced dorsal hyomandibula (Figure S5A) as reported previously in *jag1b* mutants.^12^ In addition, Jag1b is a well‐known ligand for Notch signaling upstream of *hey1*,^8^ which was also upregulated in our mutant embryos (Figure 6H,I). We found that control embryos injected with the *jag1b* MO have reduced expression of *hey1* (Figure 6L). Taken together, these data confirm that our MO‐based strategy is both reliable and effective in zebrafish embryos. Notably, we also found that treating mutant embryos with the *jag1b* MO partially prevented the phenotype, with a subset of mutants showing normal development (Figure 6K).

Next, we designed four gRNAs that target the *jag1b* gene and injected this set of CRISPR/Cas9 ribonucleoprotein complexes into the embryos (Figure S5B). We found that each gRNA resulted in the effective editing of *jag1b* transcription (Figure S5B), thus knocking out *jag1b* expression in the injected embryos (Figure 6M). Consistent with our results obtained with the *jag1b* morphants, we found that treating mutant embryos with the CRISPR/Cas9 ribonucleoprotein partially restored PA development (Figure 6K’,N). These results support our hypothesis that increased *jag1b* expression at least partially accounts for the impaired PA formation observed in *slc30a1 a*/*slc30a1b* double‐knockout embryos.

To investigate whether the inhibitory effect of *j* *ag1b* on PA formation requires the Notch signaling pathway, we measured the expression of the reporter gene *hey1* in both mutant and control embryos and found that *hey1* expression was significantly higher in mutant embryos compared to controls (Figure 6H,I). Moreover, we found that both mutants injected with a *notch2* MO and mutants treated with the Notch signaling inhibitor DAPT (which inhibits γ‐secretase) had higher pharyngeal cartilage development compared to untreated mutants (Figure S6A,B). Taken together, these findings indicate that *slc30a1a* and *slc30a1b* regulate PA differentiation via the Jagged‐Notch signaling pathway.

### 2.7 Zinc modulates PA development by targeting *snai2*‐mediated *jag1b* expression

Next, we attempted to identify the upstream regulator of *jag1b* that contributes to the abnormal PA development in mutant embryos. We, therefore, integrated the stemness markers upregulated in mutant embryos with publicly available databases containing candidate genes and/or pathways upstream of *jag1* expression. TRRUST (Transcriptional Regulatory Relationships Unraveled by Sentence‐based Text‐mining) is a manually curated database of human and mouse transcriptional regulatory networks, providing transcription factors that target regulatory pathways derived from more than 20 million articles in PubMed describing small‐scale experimental studies of transcriptional regulation.^55^ SIGNOR (SIGnaling Network Open Resource) is a public repository containing signaling information as binary causal relationships between biological entities, collected from more than 11,000 manually annotated causal relationships between proteins that participate in signal transduction.^56^

**FIGURE 7:**
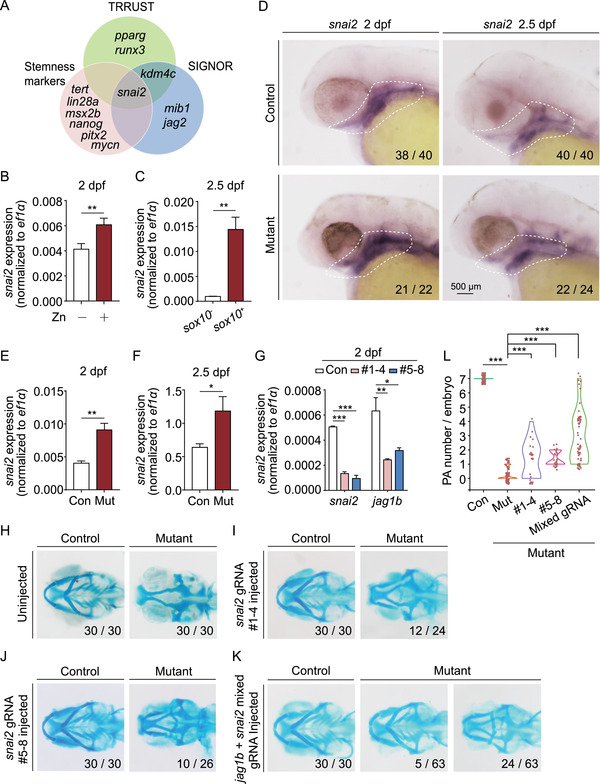
Snai2 is the upstream regulator of *jag1b*. (A) Venn diagram summarizing the overlap among genes determined by cross‐referencing the Transcriptional Regulatory Relationships Unraveled by Sentence‐based Text‐mining (TRRUST) database, SIGnaling Network Open Resource (SIGNOR) database, and stemness markers analyzed using SMART‐seq. (B) Summary of *snai2* mRNA measured in *sox10* ^+^ cells isolated from control embryos and control embryos exposed to 1 mM zinc. (C) Summary of *snai2* mRNA measured in *sox10* ^‒^ and *sox10* ^+^ cells isolated from *Tg(sox10:kikGR*) embryos. (D) in situ hybridization of *snai2* in control and mutant embryos at 2 and 2.5 dpf. (E and F) Summary of *snai2* mRNA measured in *sox10* ^+^ cells isolated from control and mutant embryos at 2 dpf (E) and 2.5 dpf (F). (G) Summary of *snai2* mRNA and *jag1b* mRNA measured in wild‐type embryos and wild‐type embryos injected with two different sets of *snai2* CRISPR/Cas9 ribonucleoprotein complexes. (H–K) Representative images of cartilage staining of control and mutant embryos. Where indicated, the embryos were injected with the *snai2* CRISPR/Cas9 ribonucleoprotein complexes (I and J) or both the *snai2* and *jag1b* CRISPR/Cas9 ribonucleoprotein complexes (K). Examples of two distinct mutant phenotypes, with the corresponding frequencies, are shown in panel (K). (L) Violin plot summarizing the number of PA pairs/embryos in control embryos and mutant embryos injected with the *snai2* CRISPR/Cas9 ribonucleoprotein complexes alone or together with the *jag1b* CRISPR/Cas9 ribonucleoprotein complexes (mixed gRNA). *p < 0.05, **p < 0.01, and ***p < 0.001

As shown in Figure 7A, *snai2* was the sole candidate gene that overlaps with upregulated stemness markers and these two databases. Using qPCR, we found that treating embryos with zinc significantly upregulated *snai2* expression (Figure 7B); moreover, we found that *snai2* is expressed robustly in *sox10* ^+^ cells, but not in *sox10* ^‒^ cells (Figure 7C). Using in situ hybridization, we also found that *snai2* is expressed throughout the PA at 2 dpf, whereas its expression is largely restricted to the ventral PA at 2.5 dpf (Figure 7D). In addition, *snai2* expression was higher in the mutants than in control embryos at both 2 and 2.5 dpf (Figure 7D). The increased expression of *snai2* in mutant *sox10* ^+^ cells compared to control *sox10* ^+^ cells was confirmed using qPCR at both 2 dpf (Figure 7E) and 2.5 dpf (Figure 7F).

Next, we designed eight gRNAs to targeted the *snai2* gene and injected two sets of four CRISPR/Cas9 ribonucleoprotein complexes in embryos (Figure S6C). We found that each set of gRNAs led to editing of *snai2* (Figure S6C), thus knocking down *snai2* expression in injected embryos (Figure 7G). Interestingly, we found that reducing *snai2* expression using either set of four CRISPR/Cas9 ribonucleoprotein complexes significantly decreased *jag1b* expression (Figure 7G) and partially restored PA formation in mutant embryos (Figure 7H–J). Moreover, co‐injecting the *snai2* and *jag1b* CRISPR/Cas9 ribonucleoprotein complexes was more effective than injecting either complex alone with respect to restoring PA development and cartilage production (Figure 7K–L). Taken together, these results indicate that *snai2* expression is regulated by zinc and that Snai2 promotes PA development via *jag1b* expression.

### 2.8 The double ZF domain in Snai2 is required for the zinc‐responsive regulation of PA development

**FIGURE 8:**
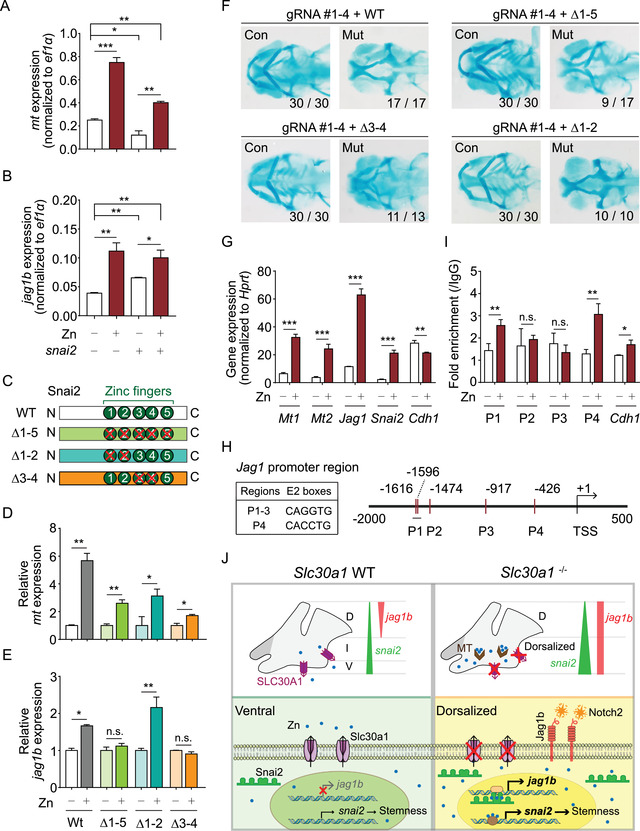
*snai2* regulates *jag1b* expression via its double zinc‐finger domain. (A and B) Summary of *mt* mRNA(A) and *jag1b* mRNA (B) measured in *sox10* ^+^ cells isolated from control embryos treated with zinc and/or injected with *snai2* mRNA. (C) Schematic diagram depicting the wild‐type (WT) Snai2 protein and three mutant Snai2 constructs lacking the indicated zinc‐finger (ZF) domains. (D and E) Summary of *mt* mRNA (D) and *jag1b* mRNA (E) measured in *sox10* ^+^ cells isolated from control embryos co‐injected with *snai2* CRISPR/Cas9 ribonucleoprotein complexes together with either WT *snai2* mRNA or the indicated mutant *snai2* mRNAs; where indicated, the embryos were treated 1 mM zinc. (F) Representative images of cartilage staining in the heads of control and mutant embryos co‐injected with *snai2* CRISPR/Cas9 ribonucleoprotein complexes together with either WT *snai2* mRNA or the indicated mutant *snai2* mRNAs. (G) Summary of *Mt1*, *Mt2*, *Snai2*, *Jag1*, and *Cdh1* mRNA measured in mouse primary mesenchymal stem cells (MSCs) cultured in the absence or presence of 10 μM zinc. (H) Schematic illustration of the *Jag1* promoter region, showing the approximate locations and sequences of the five E2 boxes. P1 through P4 indicate the primer pairs used for chromatin immunoprecipitation (ChIP) analysis, and the transcription start site (TSS) is indicated. (I) ChIP assay of mouse primary MSCs using the SNAI2 antibody for pull‐down followed by quantitative polymerase chain reaction (qPCR) using the indicated primer pairs to amplify the *Jag1* promotor region. Where indicated, the cells were cultured in the absence or presence of 10 μM zinc. The *Cdh1* promoter was used as a positive control. (J) Under normal conditions in wild‐type embryos (top left), *slc30a1* is expressed in the ventral region to balance zinc homeostasis, *jag1b* is expressed in the dorsal region, and *snai2* is expressed in the ventral pharyngeal arch (PA) more robustly than in the dorsal PA. Ventral *snai2* expression suggests a stemness state in this region (bottom left). In the absence of Slc30a1 proteins (top right), zinc accumulates both as free ions and bound to metallothionein (MT) proteins. The expression of *snai2* is increased in the ventral region, thereby expanding the pattern *jag1b* expression. In neural crest progenitor cells (bottom right), the expression of *jag1b* is upregulated via the double zinc‐finger in the Snai2 protein in a zinc‐dependent manner. The resulting increase in Jag1b activates downstream Notch signaling, arresting chondrocyte differentiation and resulting in “dorsalized” neural crest progenitor cells. *p < 0.05, **p < 0.005, ***p < 0.001, and n.s., not significant

Snai2 is a ZF‐containing transcription factor that plays a clear role in maintaining cellular plasticity and multipotency.^57^ We, therefore, examined whether the ZF domain in Snai2 plays a role in regulating *jag1b* expression. We found that exposing zebrafish embryos to 1 mM zinc significantly upregulated the zinc marker gene *mt* in *sox10* ^+^ cells (Figure 8A). Notably, although *jag1b* expression was significantly increased in both zinc‐treated embryos and *snai2*‐overexpressing embryos (Figure 8B), zinc treatment alone was more effective than overexpressing *snai2* at increasing *jag1b* expression (Figure 8B), suggesting that Snai2 upregulates *jag1b* expression via a zinc‐dependent process.

As shown in Figure S6D, the Snai2 protein is highly conserved among humans, mice, and zebrafish; the protein contains five ZF domains (ZF1–5), with ZF3 and ZF4 forming a double ZF domain. To determine which ZF domain(s) in Snai2 responds to zinc and regulates *jag1b* expression, we generated a series of mutant *Snai2* mRNAs in which the resulting proteins lack specific ZF domains (Figure 8C). We then co‐injected control embryos with the CRISPR/Cas9 ribonucleoprotein complexes (to knockdown endogenous *snai2*) together with either wild‐type *snai2* or the various ZF‐deleted *snai2* mRNAs. We found that zinc increased *mt* expression in the injected embryos (Figure 8D), consistent with zinc accumulation. Interestingly, however, zinc treatment significantly increased *jag1b* expression in the embryos expressing either wild‐type *snai2* or the Δ1‐2 *snai2*, but had no effect on *snai2* expression in embryos expressing either the Δ1‐5 or Δ3‐4 *snai2* (Figure 8E). These results indicate that the double ZF domain formed by Z3 and Z4 in Snai2 is required for mediating the effects of zinc with respect to inducing *jag1b* expression.

Interestingly, we also found that PA formation in the *slc30a1a*/*slc30a1b* double‐knockout mutant embryos was rescued by the ribonucleoprotein complexes targeting *snai2* only when we co‐injected *snai2* mRNA lacking the double ZF domain (i.e., either the Δ1‐5 or Δ3‐4 *snai2* mRNA). In contrast, co‐expressing either wild‐type *snai2* or the Δ1‐2 *snai2* failed to rescue PA formation in mutant embryos (Figure 8F). Taken together, these findings suggest that the zinc‐mediated increase in *snai2* expression inhibits PA formation by upregulating *jag1b* via the double ZF domain in Snai2.

Finally, to determine whether zinc regulates *Snai2* and *Jag1b* expression in higher vertebrates such as mammals, we measured the expression of *Mt*, *Snai2*, *Jag1*, and *Cdh1* (which is suppressed by Snai2) in mouse mesenchymal stem cells cultured in the absence or presence of 10 μM zinc. We found that zinc treatment significantly upregulated *Mt*, *Snai2*, and *Jag1*, while significantly downregulated *Cdh1* (Figure 8G). Snai2 regulates gene expression by binding to the E2 boxes (CAGGTG or CACCTG) in its target gene's promoter region.^57^ We, therefore, designed four primer sets (P1–4) corresponding to each of the five E2 boxes (with P1 corresponding to the first two E2 boxes) in the *Jag1* promoter (Figure 8H), and then performed chromatin immunoprecipitation (ChIP) analysis using a Snai2 antibody for pull‐down followed by qPCR analysis using primer sets P1–4. We found that the sequences corresponding to primer sets P1 and P4 were highly enriched. As a positive control, we also found that the E2 boxes in the *Cdh1* gene were enriched following treatment with 10 μM zinc (Figure 8I). These results indicate that zinc promotes the binding of Snai2 to the *Jag1* promoter, upregulating *Jag1* expression.

## 3 DISCUSSION

Abnormal development of the neural crest‐derived PA has been associated with a variety of congenital birth defects in humans.^58^ Although considerable progress has been made with respect to the signaling pathways that regulate PA development from multipotent NCCs, the underlying mechanisms remain poorly understood.^59^ Here, using zebrafish as a model organism, we found that the zinc transporters Slc30a1a and Slc30a1b play an essential role in NC development and PA differentiation.

The putative zinc exporter Slc30a1 is a member of the Slc30a protein family,^21^ and mice lacking *Slc30a1* are embryonic lethal.^23^ Recently, Muraina et al. reported that zebrafish in which the last 40 amino acids of Slc30a1a are deleted have altered epiboly and impaired zinc homeostasis.^24^ Here, we report that zebrafish embryos lacking both *slc30a1a* and *slc30a1b* expression fail to develop a lower jaw and have reduced pharyngeal cartilage, as well as abnormal differentiation of neural crest progenitor cells, suggesting that both Slc30a1a and Slc30a1b are essential for embryonic development, particularly with respect to the formation of the PA from the NC.

Interestingly, we found that *slc30a1a*/*slc30a1b* double‐knockout embryos have lower zinc levels compared to control embryos and are more susceptible to zinc deficiency. Consistent with this finding, *Drosophila* that lack *slc30a1* expression in the gut are also more sensitive to zinc deficiency.^25^ In this respect, it is interesting to note that our in situ hybridization analysis showed that both *slc30a1a* and *slc30a1b* are expressed in the gut of zebrafish embryos. In early zebrafish development, *slc30a1a* is expressed around the yolk syncytial layers,^24^ which provides nutritional uptake from the yolk. Therefore, it is reasonable to speculate that a loss of Slc30a1 may lead to reduced zinc absorption from both the gut and the yolk syncytial layers, thus explaining the significantly reduced systemic zinc levels in *slc30a1a*/*slc30a1b* double‐knockout embryos.

In vertebrate embryonic development, zinc is an essential trace element.^60^, ^61^ However, precisely how zinc affects development and differentiation is currently unknown. Several in vitro studies investigated the regulatory role of zinc in stem cell programming, yielding contradictory results.^16^, ^17^, ^18^, ^19^ Nevertheless, whether zinc plays a role in NC development and/or pharyngeal stemness maintaining has not been investigated. In the *slc30a1* mutants, the NC‐derived pigments developed a comparable morphology to control siblings (Figure 1G), suggesting that the phenotype of pharyngeal cartilage deficiency is not resulted from a general NC development delay.

Thus, we provide novel evidence that zinc homeostasis is a key mechanism in regulating the stemness state of the pharyngeal progenitors, and that zinc accumulation in NCCs is tightly associated with the pharyngeal stemness arrest in developing zebrafish.

Given that zinc deficiency in embryogenesis causes the abnormal development of bone and cartilage,^62^ an interesting question is whether the phenotype observed in our mutant zebrafish is due to extracellular signaling or a cell‐autologous effect. Both Edn1 and Bmp4 are important exogenous signals that govern PA development.^8^ Moreover, their downstream signals are located primarily at ventral‐intermediate PAs. Our analysis shows that the bulk gene expression in the intermediate PA and ventral‐intermediate PA is similar between mutant and control embryos. In addition, we found that the expression of *hand2*—a canonical target for both Edn1 and Bmp4^63^—is similar in the ventral PA between mutant and control embryos, suggesting that the impaired PA development in mutant embryos is likely due to a cell‐autologous effect.

Studies have shown that the graded expression of the so‐called Distal‐less‐related (Dlx) genes (including *dlx3*, *dlx4*, *dlx5*, and *dlx6*) among progenitor cells promotes the differentiation of pharyngeal chondrocytes,^45^ whereas Jag1b‐Notch2 signaling functions in the dorsal PA to inhibit differentiation and formation.^11^, ^12^ Our unbiased SMART‐seq analysis revealed that *jag1b* and *dlx4a* are significantly upregulated and downregulated, respectively, in mutant embryos, and inhibiting the Jag1b‐Notch2 pathway—but not overexpressing *dlx4a*—rescues PA development in mutant embryos. Given concerns regarding the ability of MOs to recapitulate mutant phenotypes,^64^ we used the *jag1b* and *notch2* MOs that were previously reported to produce a phenotype similar to mutant animals.^12^ We, therefore, conclude that loss of Slc30a1 proteins causes zinc overload in pharyngeal NCCs, which triggers these cells to arrest in a multipotent, dorsalized pattern.

Although the Jagged‐Notch pathway is known to function in the dorsal PA, its upstream signaling is poorly understood. We found that *snai2* is upstream of *jag1b*. In addition to serving as a marker gene for NC specification and migration, Snai2 also functions to promote cell survival and control stem cell properties in cancer cells.^57^ Interestingly, both *Snai2* overexpression and downregulation cause conditions associated with abnormal neural crest development in humans,^65^, ^66^, ^67^ suggesting that this gene plays an essential role in the development of the NC and NC‐derived tissues. However, precisely how *snai2* regulates the stemness state of NCCs in vivo is currently unknown. We found that *snai2* is robustly expressed in the ventral PA of mutant embryos and functions upstream of *jag1b* to regulate PA differentiation. Mechanistically, we found that the double ZF domain in Snai2 plays a key role in the response to zinc with respect to regulating *jag1b* during zinc accumulation. Our results, therefore, suggest a novel mechanism in which the zinc transporter Slc30a1 controls zinc homeostasis in pharyngeal NCCs, regulating *snai2* and downstream *jag1b* expression in order to determine PA differentiation.

In summary, we report that the zinc transporters Slc30a1a and Slc30a1b play an essential role in PA development in zebrafish, mediating zinc homeostasis in NC progenitor cells and regulating PA development by targeting the downstream *snai2*‐*jag1b*‐Notch axis (Figure 8J). These results underscore the importance of zinc homeostasis in multipotent NCCs, in which zinc accumulation induces stemness arrest. In addition, we report that the double ZF domain in Snai2 is critical for regulating *jag1b* expression in the presence of increased zinc. Importantly, these findings provide novel insights into the role that zinc transporters play in NC‐derived PA formation via the Snai2‐Jag1b cascade, providing compelling evidence that SLC30A1 may serve as a viable target in diagnosing and treating a wide range of congenital birth defects.

## 4 MATERIALS AND METHODS

### 4.1 Zebrafish

All zebrafish strains were maintained and bred in accordance with the guidelines of the core facilities at Zhejiang University School of Medicine and the Laboratory Animal Center, Zhejiang University.

### 4.2 Cell dissociation and flow cytometric sorting

Zebrafish embryos were digested for 30 min at 28°C in a protease solution containing 2 mg/ml collagenase (Roche) in trypsin‐EDTA (Gibco). The reaction was terminated by adding a fetal bovine serum to a final concentration of 10% (v/v). The cells were collected by centrifugation at 2000 rpm for 5 min at 4°C, resuspended in 4% fetal bovine serum in phosphate‐buffered saline (PBS), and placed on ice. To remove dead cells for flow cytometry, the cells were stained with SYTOX Blue (Invitrogen) before sorting using a FACS ARIA II SORP cell sorter (BD Biosciences). The cells were then directly homogenized in TRIzol LS reagent (Invitrogen), and total RNA was extracted using the Direct‐zol RNA kit (R2050, Zymo Research).

### 4.3 Whole‐mount in situ hybridization

in situ hybridization was performed as described previously^68^; for these experiments, mutant embryos and control siblings were mixed in a well for each reaction. All probes were cloned into pEASY‐T3 (Trans‐gene), and images were captured using an SMZ18 or AZ100 stereomicroscope (Nikon). Repeated experiments and quantitative data are presented as a fraction (X/N), in which the denominator (N) represents the total number of embryos used for in situ and numerator (X) represents the number of embryos with a given phenotype.

### 4.4 Gene knockout using CRISPR/Cas9

Target sites were designed and selected using the CHOPCHOP website (https://chopchop.cbu.uib.no/). Guide RNA (gRNA) templates were synthesized as previously described,^69^ and one or more gRNAs were mixed with Cas9 protein (CP02, PNA Bio) for microinjection into single‐cell stage embryos. The efficiency of genetically disrupting each target was determined by DNA sequencing, quantitative PCR, and/or in situ hybridization.

### 4.5 Alcian blue staining

At 4 dpf, zebrafish embryos were fixed in 4% paraformaldehyde (PFA) overnight, followed by dehydration in 75% methanol. The dehydrated embryos were then transferred to a solution containing 0.02% Alcian blue and 60 mM MgCl_2_ in ethanol for skeletal staining. The following day, the pigments were removed by incubation in 3% hydrogen peroxide for 2 h; the embryos were then rinsed in PBS and digested for 4 h in 50 mg/ml trypsin. Repeated experiments and quantitative data are presented as a fraction (X/N), in which the denominator (N) represents the total number of embryos used for Alcian blue staining and numerator (X) represents the number of embryos with a given phenotype.

### 4.6 Genotyping

For genotyping adult zebrafish, we biopsied the tail and extracted genomic for PCR analysis. We determined the Mendelian inheritance for all embryos with the same phenotype by dividing each embryo into two parts: the head was used for Alcian blue staining, and the tail was used to extract genomic DNA for genotyping. Using this approach, we matched the genotype of both *slc30a1* alleles with the corresponding phenotype, and perform rescue experiments in which we could match the genotype of each embryo with the corresponding phenotype. For in situ hybridization, we extracted genomic DNA from each embryo after in situ hybridization and performed sequencing to determine the genotype.

### 4.7 Immunofluorescence and TUNEL staining

Embryos fixed in 4% PFA were permeabilized with 0.1% Triton X‐100, rinsed several times in PBST buffer, and then blocked in 5% goat serum. For Col2 immunostaining, anti‐Collagen type II (1:100; II‐116B3, Developmental Studies Hybridoma Bank) and Cy3‐goat anti‐mouse (A0521, Beyotime) were used as the primary and secondary antibodies, respectively.^5^ For phosphohistone‐H3 (PH3) immunostaining, pH3‐Ser‐10 (sc‐8656‐R, Santa Cruz) and Cy3‐goat anti‐rabbit (A0516, Beyotime) were used as the primary and secondary antibodies, respectively. For TUNEL staining, the in situ Cell Death Detection Kit (12156792910, Roche) was used in accordance with the manufacturer's instruction. Images were captured using an FV1000 Bx61 or SpinSR IX83 confocal microscope (Olympus). At least five embryos from different treatments were used for quantification and statistical analysis.

### 4.8 Microinjection of plasmids, mRNAs, and morpholinos

The complete coding sequence of each gene was cloned and inserted into the pCS2 plasmid, which was linearized overnight and then transcribed using the mMESSAGE mMACHINE kit (AM1344, Ambion). The purified plasmid (20–30 pg/embryo) or mRNA (200–300 pg/embryo) was then injected into single‐cell stage zebrafish embryos. The sequences of the morpholinos are listed in Table S5.^53^, ^70^

### 4.9 Inductively coupled plasma mass spectrometry

ICP‐MS was performed as described previously.^69^ In brief, zebrafish embryos totaling at least 100 mg were collected in order to obtain sufficient material for subsequent analysis. The samples were transferred into acid‐washed cans for digestion, after which the residual acid was removed by evaporation and washing in ultrapure water. The digested samples were then diluted in Milli‐Q water and measured using a 7500ce ICP‐MS system (Agilent).

### 4.10 FACS analysis using a fluorescent zinc indicator

Intracellular zinc concentration was measured using the fluorescent indicator FluoZin‐3 AM (F24195, Invitrogen) as previously described.^71^ For the *Tg(sox10:kikGR)* zebrafish line, the embryos were exposed to UV light to convert green fluorescence to red fluorescence. The UV‐exposed embryos were then digested, and the cells were sorted using FACS. The sorted cells were collected and suspended in a 200 μl detection buffer containing (in mM): 5 glucose, 1 MgCl_2_, 1 NaH_2_PO_4_, 1.3 CaCl_2_, 25 HEPES, 120 NaCl, 5.4 KCl, and 1 μM FluoZin‐3 AM (pH 7.5). The cells were then incubated for 30 min at 37°C, followed by several rinses with detection buffer. The cells were then resuspended in a 1 ml detection buffer and distributed equally into three samples. Sample 1 was used to measure the normal amount of fluorescence (*F*). Note that, 50 μM TPEN (Sigma) was added to sample 2 and used to measure the minimum fluorescence (*F*_min_), and 100 μM zinc and 50 μM NaPyr (Sigma) was added to sample 3 and used to measure the maximum fluorescence (*F*_max_). All samples were incubated at 37°C for 10 min and then analyzed using FACS. The zinc concentration ([Zn]) was calculated using the following equation: [Zn] = (KD x [(*F* – *F*_min_)/(*F*_max_ – *F*)]), with a KD value of 15 for FluoZin‐3 AM.

### 4.11 SMART‐seq

The SMARTer kit was used to synthesize SMART (Switching Mechanism At the 5’ end of RNA Transcript) cDNA from total RNA extracted from *sox10*:kikGR^+^ cells using modified oligo(dT) primers and SMARTScribe Reverse Transcriptase. Amplified single‐stranded cDNA was then used as a template for long‐distance PCR to produce sufficient double‐stranded cDNA (ds‐cDNA) for library construction. These ds‐cDNAs were then fragmented by incubation at 37°C for 30 min in dsDNA fragmentase (M0348S, NEB). Library construction was initiated using fragmented cDNA, followed by paired‐end sequencing using an Illumina NovaSeq 6000 system (LC Sciences). Sample reads were aligned to the Ensembl *Danio rerio* (zebrafish) genome GRCz11 (http://www.ensembl.org/) using the HISAT package. The mapped reads of each sample were assembled using StringTie, and all transcriptomes were merged to reconstruct a comprehensive transcriptome using Perl scripts. After the final transcriptome was generated, StringTie and edgeR were used to estimate the expression levels of each transcript. StringTie was then used to calculate the FPKM (Fragments Per Kilobase of transcript per Million mapped reads) in order to quantify the mRNAs. Differentially expressed mRNAs and genes were selected based on a log2 (fold change) > 1 or < ‐1 and with *p *< 0.05 using the *R* package.

### 4.12 Isolation and culture of mouse mesenchymal stem cells

The femurs and tibias were removed from 12 wild‐type C57BL/6N mice, the ends of the bones were cut off, and the contents of the marrow were flushed in a growth medium (MUBMX‐90011, Cyagen). The cells that were flushed from the marrow were then plated in 10‐cm dishes. Note that, 48 h later, non‐adherent cells were removed carefully, and a fresh medium was added. After 72 h in culture, non‐adherent cells were again removed, and the medium was then changed daily in order to obtain a purified cell population. When cell density reached at least 80% (typically within 10 days of culture), 10 μM zinc was added to the medium; the following day, the cells were collected and used for ChIP analysis.

### 4.13 ChIP assay

ChIP was performed using the Simple ChIP Plus Enzymatic Chromatin IP Kit (9005; Cell Signaling) in accordance with the manufacturer's instructions. Immunoprecipitation was performed using magnetic beads (9006; Cell Signaling) and antibodies against SNAI2 (sc‐166902; Santa Cruz Biotechnology). Recovered DNA fragments were then used directly for quantitative RT‐PCR analysis using primers corresponding to the *Jag1* promoter (Table S6).

### 4.14 Reverse transcription, quantitative PCR, and semi‐quantitative PCR

Total mRNA was isolated from whole zebrafish embryos and mouse mesenchymal stem cells (MSCs) using TRIzol reagent (Invitrogen), and cDNA was synthesized using M‐MLV reverse transcriptase (M1701, Promega). Real‐time PCR was performed using a two‐step quantitative RT‐PCR kit (RR047A, Takara Bio), and target gene expression was normalized to *ef1α* mRNA (for zebrafish) or *Hprt* mRNA (for MSCs). For semi‐quantitative PCR, 27 amplification cycles were used, and target gene expression was normalized to *β‐actin* mRNA.

### 4.15 Quantification and statistical analysis

Except where indicated otherwise, all summary data are presented as the mean ± the standard error of the mean from at least three independent experiments. Differences between the two groups were analyzed using the two‐tailed Student's *t*‐test as described previously.^69^ For enrichment analysis, the hypergeometric test was used to calculate the *p*‐value. For box plots, we analyzed the data as previously reported.^32^ In brief, to test whether the log2 fold‐change values for each group of genes were significantly different from zero, we used the Shapiro–Wilk test for normality to determine whether a one‐sample Student's *t*‐test or Wilcoxon signed‐rank test was appropriate; we then calculated the *p*‐value for each group accordingly. Differences with a *p*‐value < 0.05 were considered statistically significant.

## CONFLICT OF INTEREST

The authors declare to have no conflict of interest.

## AUTHOR CONTRIBUTIONS

Fudi Wang, Junxia Min, and Zhidan Xia conceived the project and designed the experiments. Zhidan Xia, Xinying Bi, Junxia Min, Fudi Wang, Lothar Rink, and Pengfei Xu wrote and revised the manuscript. Zhidan Xia, Xinying Bi, Jiayu Wei, Sisi Yang, and Xiu Yang performed the experiments. Zhidan Xia, Xinying Bi, Fudi Wang, and Junxia Min analyzed and interpreted the experimental data. Zhidan Xia and Zijun Song performed the bioinformatics analysis. Fudi Wang and Junxia Min supervised the study.

## ETHICS APPROVAL

All animal studies were approved by the Institutional Animal Care and Use Committee of the Laboratory Animal Center, Zhejiang University.

## Supporting information

Supporting information

## Data Availability

The SMART‐seq data generated in this study are available at NCBI's Gene Expression Omnibus GEO database (https://www.ncbi.nlm.nih.gov/geo/query/acc.cgi; accession # GSE173696).
