# Supplementary material for: Metal transporter Slc30a1 controls pharyngeal neural crest differentiation via the zinc‐Snai2‐Jag1 cascade
Source: MedComm (2020). 2021 Sep 27;2(4):778–97. doi: 10.1002/mco2.91 (PMC8706747; doi:10.1002/mco2.91)
Supplement: Supplementary file 1 — Supporting information [file MCO2-2-778-s001.docx]

**SUPPLEMENTAL FILES**

**Metal Transporter Slc30a1 controls pharyngeal neural crest differentiation via the zinc-Snai2-Jag1 cascade**

Zhidan Xia, Xinying Bi, Sisi Yang, Xiu Yang, Zijun Song, Jiayu Wei, Pengfei Xu, Lothar Rink, Junxia Min, Fudi Wang


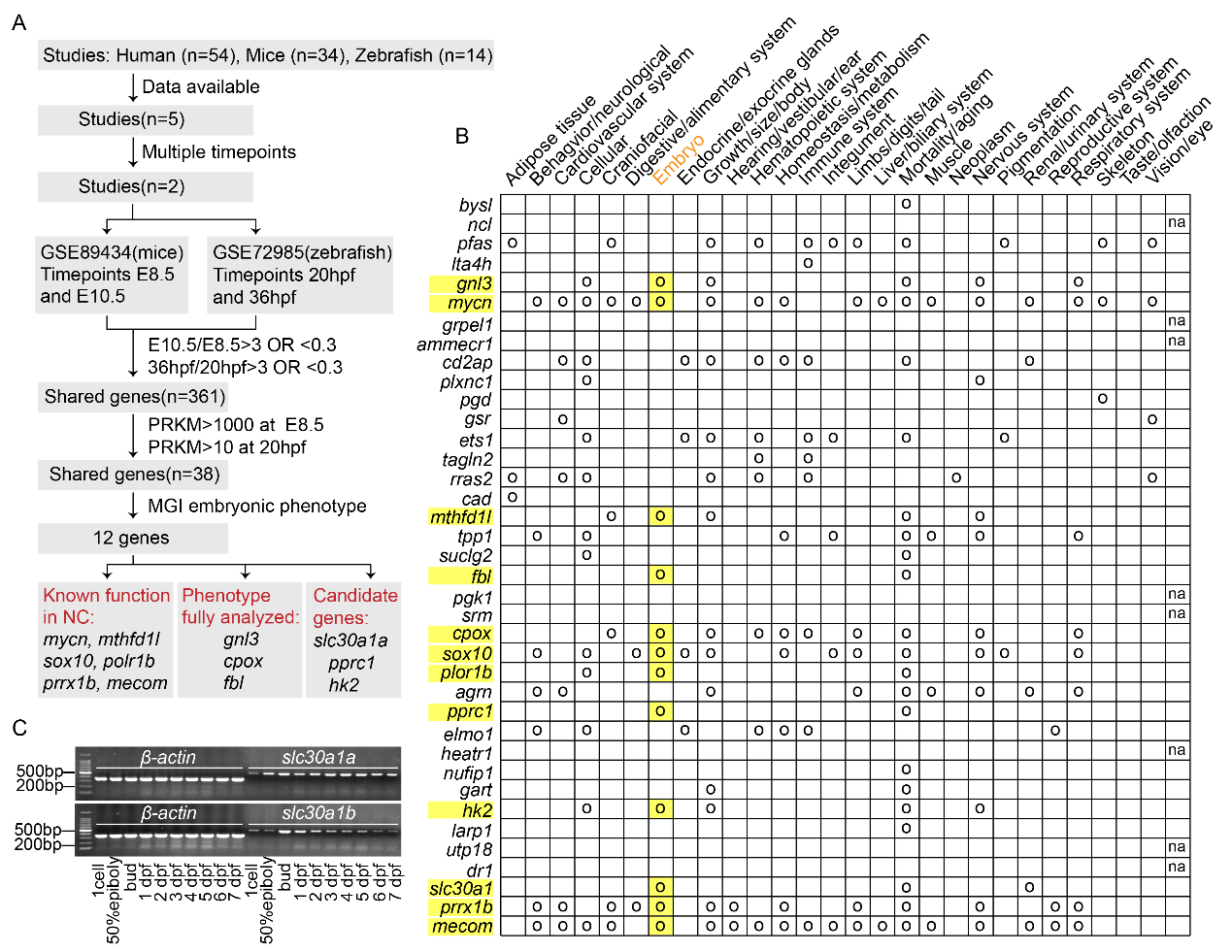


**Figure S1. Multipotent NC-related gene screening.**

(A) Strategy for screening candidate genes involved in NC development and PA patterning, revealing 12 genes with an embryonic phenotype, including 3 candidate genes.

(B) Phenotype overview from the Mouse Genome Informatics (MGI) database; the 12 genes with an embryonic phenotype are highlighted in yellow.

(C) Semiquantitative PCR analysis of *slc30a1a* and *slc30a1b* mRNA at the indicated stages in wild-type zebrafish embryos. *β-actin* mRNA was included as a control.


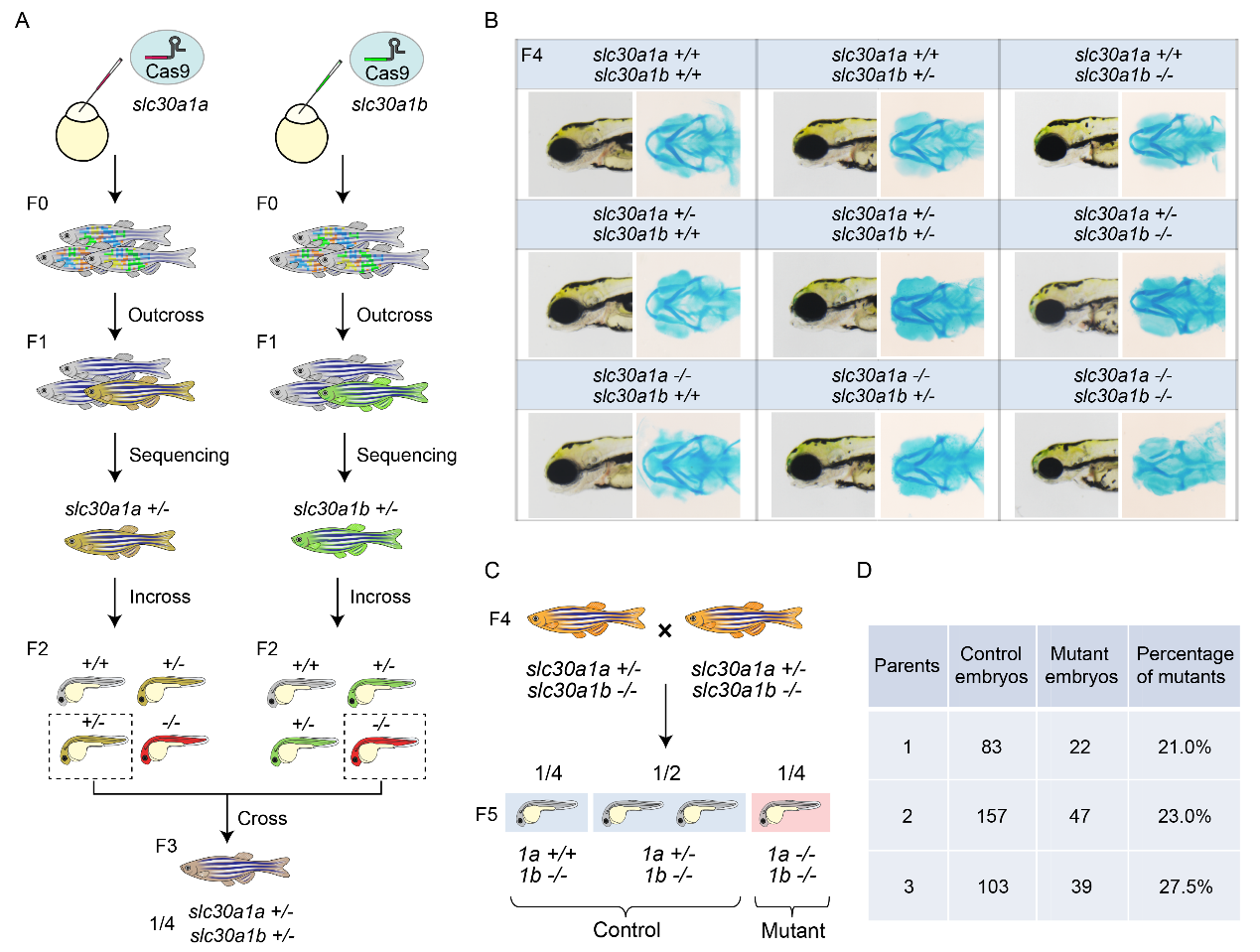


**Figure S2. Screening of *slc30a1a* and *slc30a1b* knockout mutants.**

(A) Flow chart depicting the strategy for screening *slc30a1a* and *slc30a1b* double heterozygous.

(B) Representative images of embryos and cartilage staining in the heads of embryos with the indicated *slc30a1a* and *slc30a1b* alleles.

(C) Flow chart depicting the strategy for crossing and screening *slc30a1a* and *slc30a1b* double knockout mutants.

(D) Distribution of control and mutant embryos obtained from three parental pairs.


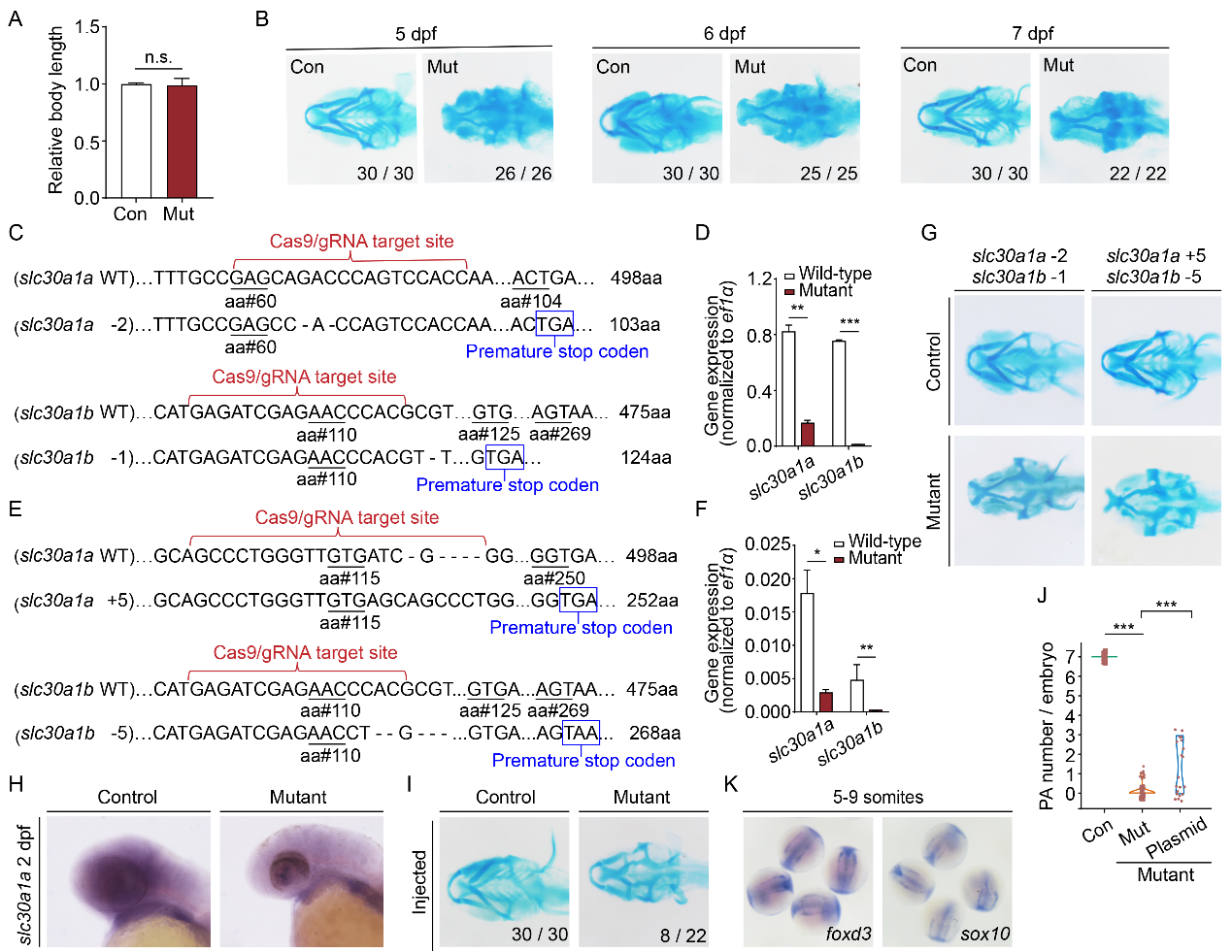


**Figure S3. Strategy for knocking out the *slc30a1a* and *slc30a1b* genes, and expression profiling of early-NCC markers.**

(A) Summary of the relative body length measured in control and mutant embryos at 4 dpf.

(B) Representative images of cartilage staining in the heads of control embryos and *slc30a1* double mutant embryos at 5-7 dpf.

(C-F) Strategy used to generate *slc30a1a* and *slc30a1b* knockout zebrafish lines (C, E) and the resulting respective knockout efficiency (D, F). The gRNA target site for each gene is shown in red, and the resulting premature stop codons are indicated. The gene editing strategy is indicated for each gene (e.g., -2 and +5 refer to a 2-bp deletion and a 5-bp insertion, respectively).

(G) Representative images of cartilage staining in the heads of control embryos and *slc30a1* double mutant embryos generated using the indicated editing strategies.

(H) Whole-mount *in situ* hybridization of *slc30a1a* mRNA in control and mutant embryos.

(I) Representative images of cartilage staining in the heads of a control embryo and a mutant embryo injected with PCS2-*slc30a1a* and PCS2-*slc30a1b* mixed plasmids. Eight embryos in total 22 embryos were partial rescued by these plasmids.

(J) Violin plot summarizing the number of PA pairs/embryo in control embryos, mutant embryos, and mutant embryos injected with *slc30a1a* and *slc30a1b* plasmids

(K) Example images of zebrafish embryos stained for *foxd3* and *sox10* mRNA using whole-mount *in situ* hybridization at the 5-9 somite stage. Note that both images include both control and *slc30a1a*/*slc30a1b* double-knockout zebrafish embryos, which cannot be distinguished at this stage. Each image is representative of 60 embryos.

**p*<0.05, ***p*<0.01, and ****p*<0.001.


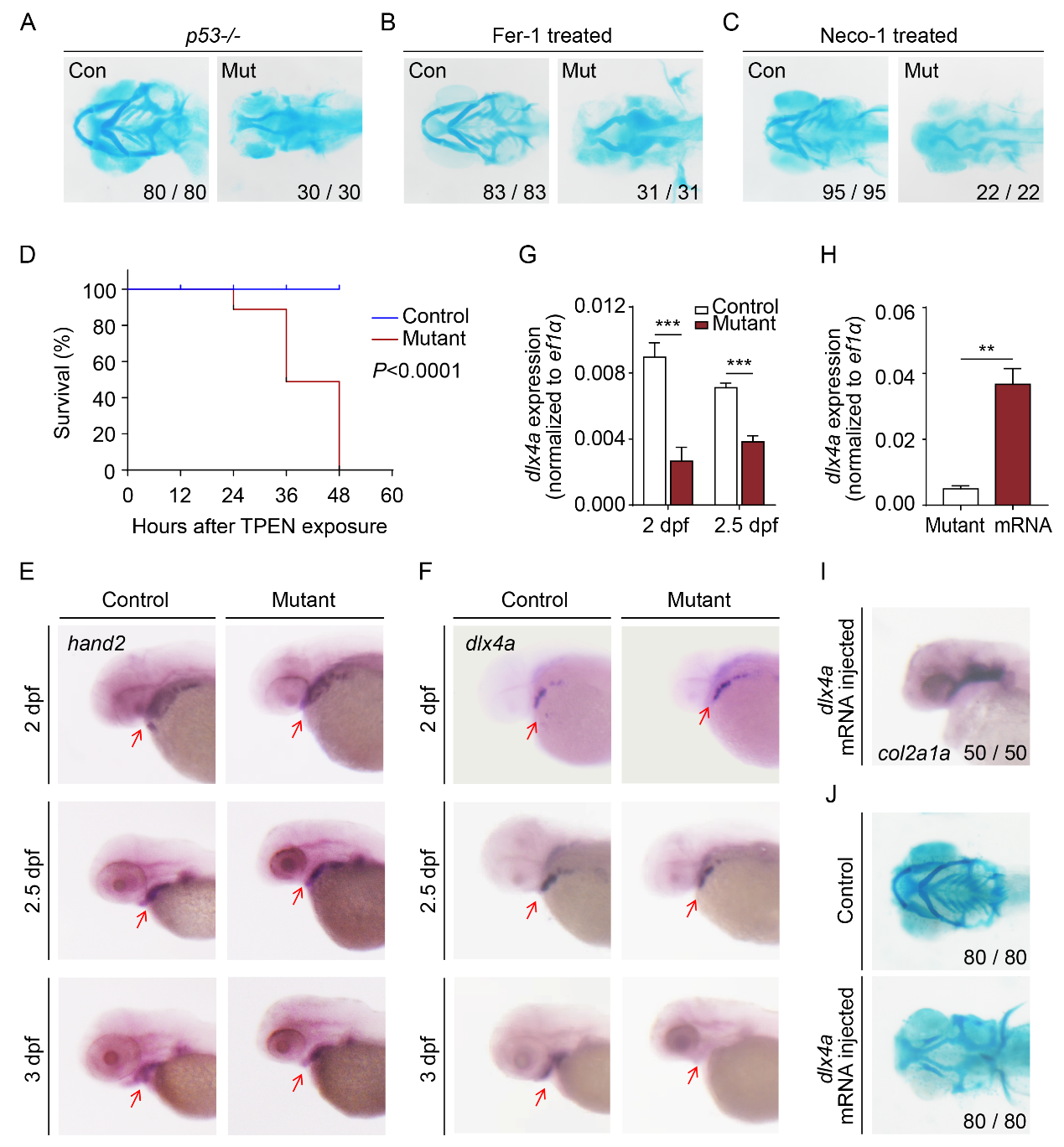


**Figure S4. Analysis of compound treatment, and expression of *hand2* and *dlx4a*.**

(A-C) Representative images of cartilage staining in the heads of control embryos and *slc30a1* double mutant embryos with a *p53* knockout background (A), or treated with Fer1 (10 μM, B) or Nec1 (100 μM, C) from 1-4 dpf.

(D) Kaplan-Meier survival analysis of control and mutant embryos treated with the cell-permeable zinc chelator TPEN (50 μM).

(E-F) Whole-mount *in situ* hybridization of *hand2* mRNA (E) and *dlx4a* mRNA (F) measured in control and mutant embryos.

(G) Summary of *dlx4a* mRNA measured in *sox10*^+^ cells isolated from control and mutant embryos.

(H) Summary of *dlx4a* mRNA measured in mutant embryos and in mutant embryos injected with *dlx4a* mRNA.

(I) Whole-mount *in situ* hybridization of *col2a1a* in a mutant embryo at 2.5 dpf injected with *dlx4a* mRNA.

(J) Representative images of cartilage staining in the heads of a control embryo and a mutant embryo injected with *dlx4a* mRNA.

***p*<0.01 and ****p*<0.001.


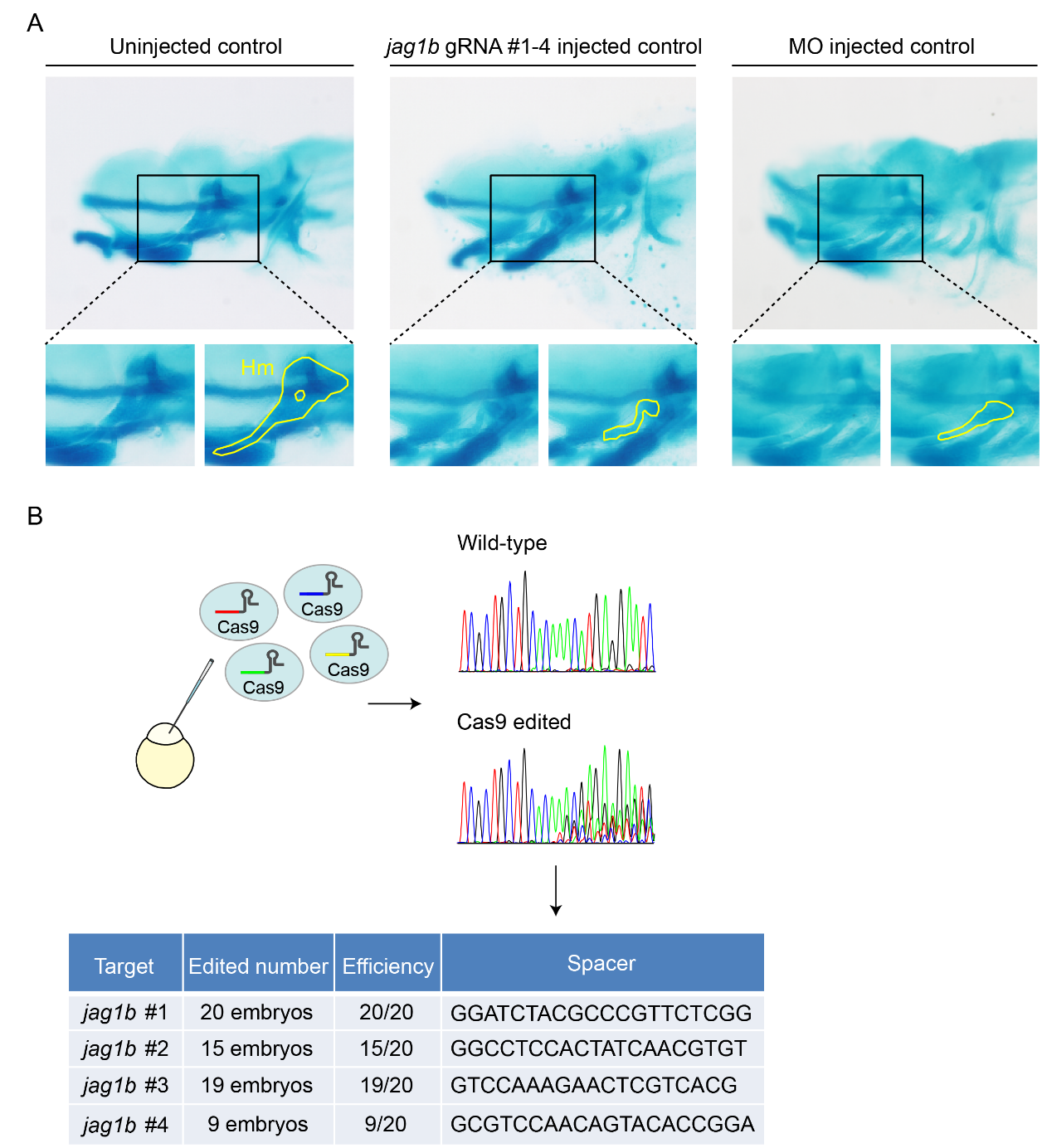


**Figure S5. Morphological analysis of pharyngeal cartilage, detection of Notch signaling, and genetic knockout of *jag1b* using CRISPR/Cas9 ribonucleoprotein complexes.**

(A) Representative images of cartilage staining in the heads of a control embryo, an embryo injected with a set of four CRISPR/Cas9 ribonucleoprotein complexes or a *jag1b* MO. All images show a lateral view. The magnified images show the dorsal hyomandibular area encircled in a yellow line.

(B) Evaluation of the knockout efficiency of *jag1b* using four CRISPR/Cas9 ribonucleoprotein complexes. A total of 20 embryos were sequenced individually. An example sequencing run shows a clear single peak near the target in a wild-type embryo and a disordered peak in an edited embryo. Editing efficiency was calculated as the number of successfully edited embryos out of 20 injected embryos.


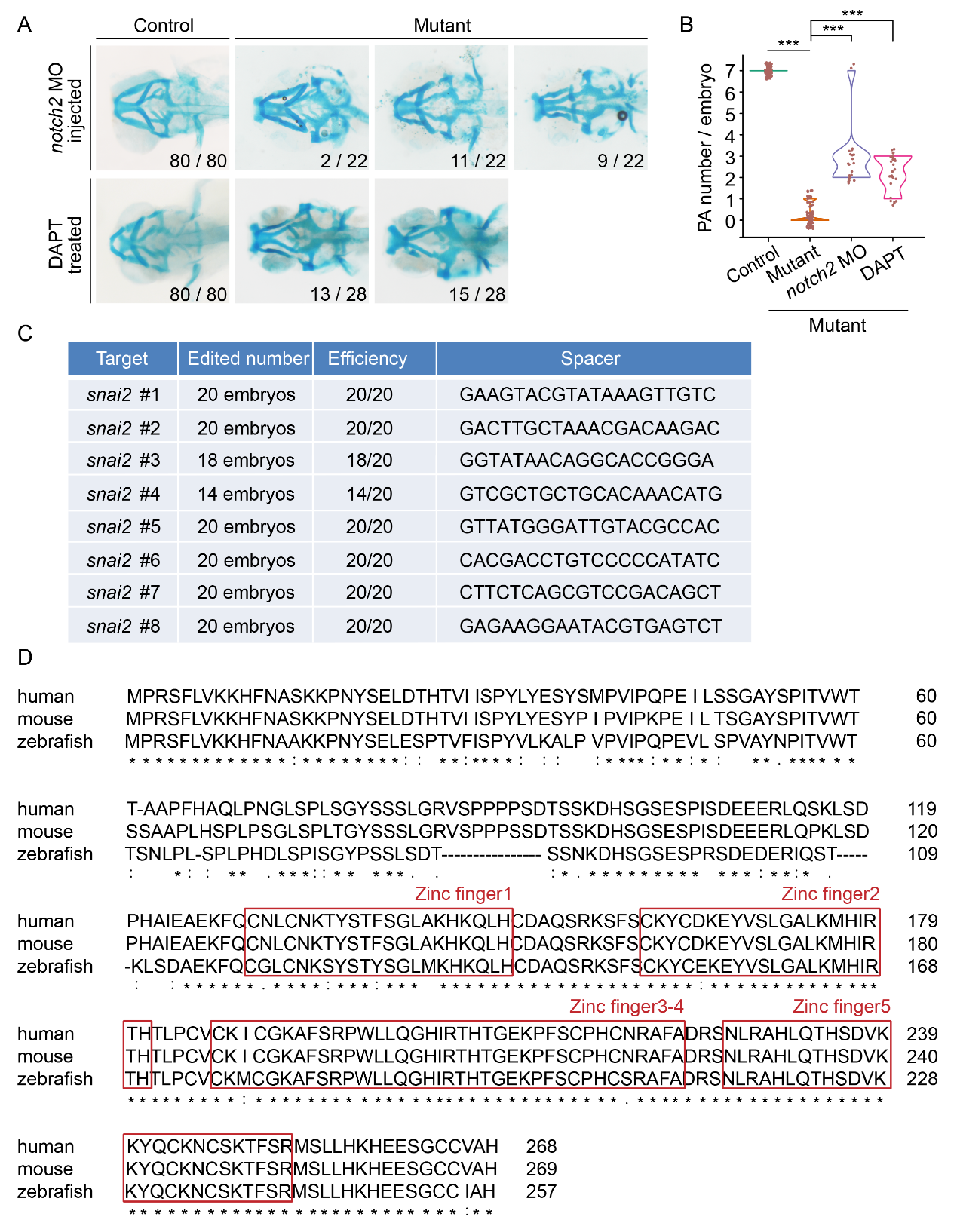


**Figure S6. Knockout efficiency of *snai2* and sequence alignment of the human, mouse, and zebrafish SNAI2 proteins.**

(A) Representative images of head cartilage staining in control and mutant embryos injected with the *notch* MO or treated with the Notch signaling inhibitor DAPT (100 μM, overnight).

(B) Violin plot summarizing the number of PA pairs/embryo in control embryos, mutant embryos, and mutant embryos injected with the *notch2* MO or treated with DAPT.

***p*<0.01, ****p*<0.001, and n.s., no significance (*p*>0.05).

(C) Evaluation of the efficiency of knocking down *snai2* using eight CRISPR/Cas9 ribonucleoprotein complexes. Editing efficiency was calculated as the number of successfully edited embryos out of 20 injected embryos.

(D) Sequence alignment of the human, mouse, and zebrafish SNAI2 proteins. The five zinc-finger (ZF) domains, including the ZF3-4 double zinc-finger domain, are indicated by red boxes.

***p*<0.01 and ****p*<0.001.

**Table S1. Summary of significantly enriched GO terms that were unique for controlling development from 2 dpf to 2.5 dpf (bold terms were selected for Figure 4A) and mutant development from 2 dpf to 2.5 dpf (bold terms were selected for Figure 4B).**

| GO_Term | S gene number | *p*-Value |
| --- | --- | --- |
| Control |  |  |
| structural constituent of eye lens | 25 | 3.87638132703927E-09 |
| oxygen transporter activity | 13 | 4.85471883916427E-08 |
| oxygen transport | 13 | 4.85471883916427E-08 |
| oxygen binding | 13 | 4.85471883916427E-08 |
| lens development in camera-type eye | 26 | 2.05653816864881E-07 |
| ionotropic glutamate receptor activity | 15 | 2.17582886263834E-07 |
| membrane | 869 | 1.46679922263804E-06 |
| synapse | 47 | 1.55705268167772E-06 |
| hemoglobin complex | 10 | 1.62230491817945E-06 |
| integral component of membrane | 780 | 0.0000122302373116723 |
| extracellular space | 126 | 0.0000122782181429804 |
| plasma membrane | 253 | 0.0000125660331863342 |
| postsynaptic membrane | 27 | 0.0000395030389545026 |
| neuronal cell body | 15 | 0.0000673079698009227 |
| structural molecule activity | 36 | 0.0000796159663941642 |
| ion transport | 87 | 0.0000971664883425705 |
| presynaptic membrane | 13 | 0.000104281563642505 |
| visual perception | 33 | 0.000111297309316472 |
| myosin complex | 18 | 0.000142507091921429 |
| motor activity | 19 | 0.000304210571374308 |
| organic acid binding | 6 | 0.000316566265371409 |
| haptoglobin binding | 6 | 0.000316566265371409 |
| haptoglobin-hemoglobin complex | 6 | 0.000316566265371409 |
| sarcomere organization | 16 | 0.000320377904190594 |
| axon extension | 12 | 0.000335566646979424 |
| cell surface | 29 | 0.000361102222554699 |
| regulation of synaptic transmission | 10 | 0.000363410458251057 |
| glutamate receptor activity | 9 | 0.00041815913299148 |
| ion channel activity | 45 | 0.000464271662586446 |
| macrophage differentiation | 6 | 0.00062869894632102 |
| cardiac muscle tissue development | 8 | 0.000699278219068122 |
| peripheral nervous system neuron axonogenesis | 7 | 0.000724897823240145 |
| dendrite development | 7 | 0.000724897823240145 |
| neurotrophin receptor activity | 4 | 0.000786910320391356 |
| kainate selective glutamate receptor complex | 4 | 0.000786910320391356 |
| neurotrophin binding | 4 | 0.000786910320391356 |
| cellular response to nerve growth factor stimulus | 4 | 0.000786910320391356 |
| regulation of microtubule depolymerization | 4 | 0.000786910320391356 |
| kainate selective glutamate receptor activity | 4 | 0.000786910320391356 |
| protein binding | 50 | 0.000847697691267357 |
| guanylate kinase activity | 3 | 0.00151107701144126 |
| endocardial cushion morphogenesis | 3 | 0.00151107701144126 |
| negative regulation of coagulation | 3 | 0.00151107701144126 |
| synaptic transmission, glutamatergic | 8 | 0.00152508618471958 |
| actin filament binding | 38 | 0.00176046582099165 |
| alpha-amino-3-hydroxy-5-methyl-4-isoxazole propionate selective glutamate receptor activity | 4 | 0.00214515501594192 |
| cell junction | 40 | 0.00215623772177231 |
| peroxidase activity | 9 | 0.00231348753433602 |
| cholesterol binding | 10 | 0.00231604845472511 |
| hydrogen peroxide catabolic process | 7 | 0.00262650378644746 |
| embryonic hemopoiesis | 10 | 0.00298595528401224 |
| transition metal ion binding | 5 | 0.00304001254340891 |
| cardioblast differentiation | **5** | **0.00304001254340891** |
| motor neuron axon guidance | 13 | 0.00415206793162815 |
| atrioventricular canal development | 6 | 0.0045351157390745 |
| epidermal growth factor receptor binding | 4 | 0.0045502381318773 |
| detection of mechanical stimulus involved in sensory perception | 5 | 0.00504944054098222 |
| endocardial cushion formation | 3 | 0.00552449522315663 |
| proximal convoluted tubule development | 3 | 0.00552449522315663 |
| keratinocyte differentiation | 3 | 0.00552449522315663 |
| positive regulation of blood vessel endothelial cell proliferation involved in sprouting angiogenesis | 3 | 0.00552449522315663 |
| neuron projection guidance | 3 | 0.00552449522315663 |
| otolith formation | 3 | 0.00552449522315663 |
| neural tube development | 3 | 0.00552449522315663 |
| response to lipopolysaccharide | 8 | 0.00693913940862623 |
| regulation of ion transmembrane transport | 24 | 0.00776684987930742 |
| epithelium development | 4 | 0.00827662124870066 |
| positive regulation of G1/S transition of mitotic cell cycle | 4 | 0.00827662124870066 |
| calcium ion transport | 17 | 0.00849914835666898 |
| metalloendopeptidase activity | 21 | 0.00857874284370397 |
| alpha-amino-3-hydroxy-5-methyl-4-isoxazolepropionic acid selective glutamate receptor complex | 7 | 0.00932696105719732 |
| endocrine pancreas development | 13 | 0.00957811840592304 |
| regulation of signal transduction | 9 | 0.0101068912625227 |
| voltage-gated ion channel activity | 24 | 0.0104045156755939 |
| myofibril assembly | 9 | 0.0123767914339922 |
| response to drug | 6 | 0.0124359413299509 |
| transmitter-gated ion channel activity | 3 | 0.0126308725773046 |
| positive regulation of exocytosis | 3 | 0.0126308725773046 |
| auditory receptor cell differentiation | 3 | 0.0126308725773046 |
| positive regulation of Arp2/3 complex-mediated actin nucleation | 3 | 0.0126308725773046 |
| granulocyte chemotaxis | 3 | 0.0126308725773046 |
| coronary vasculature development | 3 | 0.0126308725773046 |
| extracellular-glycine-gated ion channel activity | 3 | 0.0126308725773046 |
| hypotonic salinity response | 3 | 0.0126308725773046 |
| neuronal stem cell maintenance | 3 | 0.0126308725773046 |
| hemidesmosome assembly | 3 | 0.0126308725773046 |
| cyclin-dependent protein kinase 5 holoenzyme complex | 3 | 0.0126308725773046 |
| negative regulation of exocytosis | 3 | 0.0126308725773046 |
| response to cAMP | 3 | 0.0126308725773046 |
| NADH dehydrogenase complex | 2 | 0.0131730360644467 |
| glial cell migration | 2 | 0.0131730360644467 |
| phosphatidylcholine metabolic process | 2 | 0.0131730360644467 |
| plasma membrane repair | 2 | 0.0131730360644467 |
| regulation of striated muscle contraction | 2 | 0.0131730360644467 |
| cellular response to temperature stimulus | 2 | 0.0131730360644467 |
| low-density lipoprotein particle receptor binding | 2 | 0.0131730360644467 |
| late distal convoluted tubule development | 2 | 0.0131730360644467 |
| positive regulation of double-strand break repair | 2 | 0.0131730360644467 |
| xylosyltransferase activity | 2 | 0.0131730360644467 |
| glutathione dehydrogenase (ascorbate) activity | 2 | 0.0131730360644467 |
| positive regulation of T cell receptor signaling pathway | 2 | 0.0131730360644467 |
| protein xylosyltransferase activity | 2 | 0.0131730360644467 |
| purine nucleotide metabolic process | 2 | 0.0131730360644467 |
| positive regulation of release of sequestered calcium ion into cytosol | 2 | 0.0131730360644467 |
| sepiapterin reductase activity | 2 | 0.0131730360644467 |
| phosphatidylinositol 3-kinase complex, class IA | 2 | 0.0131730360644467 |
| regulation of mitosis | 4 | 0.0135552867241493 |
| peptidase activity | 57 | 0.0141991925838926 |
| voltage-gated calcium channel activity | 12 | 0.0152660833684765 |
| collagen catabolic process | 7 | 0.0154310103039071 |
| perinuclear region of cytoplasm | 14 | 0.0162644763551034 |
| sarcoplasmic reticulum | 5 | 0.0163065571722758 |
| protein binding involved in cell-cell adhesion | 5 | 0.0163065571722758 |
| calcium-dependent phospholipid binding | 13 | 0.0170054298406713 |
| cell morphogenesis | 12 | 0.0176350735479511 |
| voltage-gated calcium channel complex | 10 | 0.0182607438234667 |
| neuron apoptotic process | 4 | 0.0205654695113093 |
| determination of digestive tract left/right asymmetry | 4 | 0.0205654695113093 |
| microtubule associated complex | 4 | 0.0205654695113093 |
| cellular response to retinoic acid | 4 | 0.0205654695113093 |
| synaptic vesicle | 13 | 0.0220536920287114 |
| posterior lateral line neuromast development | 5 | 0.0221922778888768 |
| macrophage chemotaxis | 5 | 0.0221922778888768 |
| L-amino acid transmembrane transporter activity | 5 | 0.0221922778888768 |
| Schwann cell development | 3 | 0.0231165901386673 |
| Schwann cell differentiation | **3** | **0.0231165901386673** |
| regulation of cell motility | 3 | 0.0231165901386673 |
| cell dedifferentiation | 3 | 0.0231165901386673 |
| proepicardium development | 3 | 0.0231165901386673 |
| T-tubule | 3 | 0.0231165901386673 |
| adenylate cyclase-inhibiting G-protein coupled receptor signaling pathway | 3 | 0.0231165901386673 |
| positive regulation of cell differentiation | **3** | **0.0231165901386673** |
| olfactory nerve formation | 3 | 0.0231165901386673 |
| receptor antagonist activity | 3 | 0.0231165901386673 |
| olfactory placode morphogenesis | 3 | 0.0231165901386673 |
| positive regulation of synapse assembly | 3 | 0.0231165901386673 |
| Z disc | 15 | 0.0246190415120457 |
| response to bacterium | 9 | 0.0253360146319230 |
| collagen fibril organization | 6 | 0.0269473493725596 |
| tight junction | 13 | 0.0281392922305777 |
| protein localization to plasma membrane | 10 | 0.0288844398547813 |
| calcium-dependent cell-cell adhesion via plasma membrane cell adhesion molecules | 7 | 0.0292006156860349 |
| anchored component of membrane | 7 | 0.0292006156860349 |
| adenosine deaminase activity | 4 | 0.0294317717800681 |
| myofibril | 4 | 0.0294317717800681 |
| actin filament depolymerization | 4 | 0.0294317717800681 |
| hydrolase activity, acting on carbon-nitrogen (but not peptide) bonds, in linear amides | 4 | 0.0294317717800681 |
| neurotransmitter secretion | 4 | 0.0294317717800681 |
| actin cortical patch localization | 4 | 0.0294317717800681 |
| myoblast fusion | 4 | 0.0294317717800681 |
| cytoskeletal protein binding | 14 | 0.0294812115034443 |
| neural crest cell development | 9 | 0.0296873478065995 |
| heart development | 34 | 0.0330785855233794 |
| DNA-templated transcription, initiation | 10 | 0.0332383888498397 |
| metallocarboxypeptidase activity | 6 | 0.0335549949908204 |
| cupric reductase activity | 2 | 0.0364969541704576 |
| negative regulation of interleukin-8 secretion | 2 | 0.0364969541704576 |
| sulfurtransferase activity | 2 | 0.0364969541704576 |
| mesoderm migration involved in gastrulation | 2 | 0.0364969541704576 |
| dense core granule exocytosis | 2 | 0.0364969541704576 |
| carbon dioxide transport | 2 | 0.0364969541704576 |
| positive regulation of interleukin-2 production | 2 | 0.0364969541704576 |
| positive regulation of myeloid cell differentiation | 2 | 0.0364969541704576 |
| positive regulation of prostaglandin biosynthetic process | 2 | 0.0364969541704576 |
| regulation of developmental pigmentation | 2 | 0.0364969541704576 |
| positive regulation of T-helper 1 cell differentiation | 2 | 0.0364969541704576 |
| digestion | 2 | 0.0364969541704576 |
| cellular response to glucocorticoid stimulus | 2 | 0.0364969541704576 |
| prostate gland development | 2 | 0.0364969541704576 |
| alpha-beta T cell differentiation | 2 | 0.0364969541704576 |
| response to reactive oxygen species | 2 | 0.0364969541704576 |
| leukocyte migration | 2 | 0.0364969541704576 |
| ferric iron import across plasma membrane | 2 | 0.0364969541704576 |
| copper ion import | 2 | 0.0364969541704576 |
| positive regulation of neutrophil apoptotic process | 2 | 0.0364969541704576 |
| negative regulation of T-helper 2 cell differentiation | 2 | 0.0364969541704576 |
| neutrophil clearance | 2 | 0.0364969541704576 |
| cartilage condensation | **2** | **0.0364969541704576** |
| myoblast migration involved in skeletal muscle regeneration | 2 | 0.0364969541704576 |
| axonal growth cone | 2 | 0.0364969541704576 |
| ventricular cardiac muscle tissue morphogenesis | 2 | 0.0364969541704576 |
| regulation of cardiac muscle contraction by calcium ion signaling | 2 | 0.0364969541704576 |
| negative regulation of phospholipase A2 activity | 2 | 0.0364969541704576 |
| regulation of interleukin-1 production | 2 | 0.0364969541704576 |
| response to interleukin-1 | 2 | 0.0364969541704576 |
| transferase activity, transferring acyl groups, acyl groups converted into alkyl on transfer | 2 | 0.0364969541704576 |
| ferric-chelate reductase (NADPH) activity | 2 | 0.0364969541704576 |
| choline transmembrane transporter activity | 2 | 0.0364969541704576 |
| prominosome | 2 | 0.0364969541704576 |
| formin-nucleated actin cable assembly | 2 | 0.0364969541704576 |
| L-aspartate transmembrane transporter activity | 3 | 0.0370413541437595 |
| sarcomerogenesis | 3 | 0.0370413541437595 |
| hair cell differentiation | 3 | 0.0370413541437595 |
| store-operated calcium entry | 3 | 0.0370413541437595 |
| detection of light stimulus involved in visual perception | 3 | 0.0370413541437595 |
| glycosaminoglycan biosynthetic process | 3 | 0.0370413541437595 |
| adenosine to inosine editing | 3 | 0.0370413541437595 |
| macromolecular complex assembly | 3 | 0.0370413541437595 |
| aspartate transport | 3 | 0.0370413541437595 |
| regulation of leukocyte migration | 3 | 0.0370413541437595 |
| extracellular-glycine-gated chloride channel activity | 3 | 0.0370413541437595 |
| positive regulation of cell migration involved in sprouting angiogenesis | 3 | 0.0370413541437595 |
| neutral amino acid transport | 3 | 0.0370413541437595 |
| double-stranded RNA adenosine deaminase activity | 3 | 0.0370413541437595 |
| L-glutamate transport | 3 | 0.0370413541437595 |
| tRNA-specific adenosine deaminase activity | 3 | 0.0370413541437595 |
| response to radiation | 3 | 0.0370413541437595 |
| malate-aspartate shuttle | 3 | 0.0370413541437595 |
| netrin receptor activity | 3 | 0.0370413541437595 |
| melanosome | 5 | 0.0376865423043644 |
| clathrin coat assembly | 5 | 0.0376865423043644 |
| cell fate specification | 5 | 0.0376865423043644 |
| cell-cell adhesion | 16 | 0.0378346394745220 |
| heart contraction | 13 | 0.0394384721999769 |
| camera-type eye photoreceptor cell differentiation | 4 | 0.0402250593995495 |
| beta-amyloid binding | 4 | 0.0402250593995495 |
| vascular endothelial growth factor signaling pathway | 4 | 0.0402250593995495 |
| caveola | 4 | 0.0402250593995495 |
| positive regulation of protein kinase B signaling | 4 | 0.0402250593995495 |
| response to activity | 4 | 0.0402250593995495 |
| L-glutamate transmembrane transporter activity | 4 | 0.0402250593995495 |
| peptide catabolic process | 4 | 0.0402250593995495 |
| cell-cell adhesion mediated by cadherin | 6 | 0.0411258774359899 |
| synaptic vesicle endocytosis | 6 | 0.0411258774359899 |
| sensory perception of sound | 8 | 0.0413288094782566 |
| developmental pigmentation | 7 | 0.0419516376068343 |
| vasculogenesis | 10 | 0.0432800779991933 |
| dendrite | 21 | 0.0437666374272984 |
| blood vessel development | 13 | 0.0438306889132126 |
| neuron projection | 29 | 0.0450496414694622 |
| cholesterol transporter activity | 5 | 0.0473983119099086 |
| negative regulation of mitotic cell cycle | 5 | 0.0473983119099086 |
| response to unfolded protein | 5 | 0.0473983119099086 |
| Mutant |  | ***P* Value (<0.005)** |
| metal ion binding | **302** | **0.0000248612175945961** |
| nucleus | 358 | 0.000223473569165189 |
| positive regulation of transcription from RNA polymerase II promoter | 50 | 0.000319500369737602 |
| growth factor binding | 9 | 0.000319541363443032 |
| phosphatidylinositol 3-kinase binding | 5 | 0.00103485561944594 |
| central nervous system myelination | 4 | 0.00120817007349658 |
| pattern specification process | 7 | 0.00126385835085863 |
| insulin receptor signaling pathway | 7 | 0.00169155629030604 |
| pronephros development | 14 | 0.00186856828032711 |
| regulation of extracellular matrix organization | 3 | 0.0019666810722016 |
| inositol 1,4,5-trisphosphate-sensitive calcium-release channel activity | 3 | 0.0019666810722016 |
| protein tyrosine kinase collagen receptor activity | 3 | 0.0019666810722016 |
| regulation of cell-matrix adhesion | 3 | 0.0019666810722016 |
| kinesin complex | 12 | 0.00197716455730235 |
| regulation of cell migration | **12** | **0.00197716455730235** |
| transcription regulatory region DNA binding | 16 | 0.0021027089897917 |
| transmembrane receptor protein serine/threonine kinase activity | 6 | 0.0029497903931297 |
| Wnt signaling pathway | 21 | 0.00319437669397116 |
| kidney development | 8 | 0.00319738176622675 |
| retinal ganglion cell axon guidance | 9 | 0.00324866727790663 |
| sprouting angiogenesis | 15 | 0.00345128859044941 |
| notochord morphogenesis | 5 | 0.00365356862543886 |
| face morphogenesis | **4** | **0.00381285982319801** |
| regulation of JNK cascade | 4 | 0.00381285982319801 |
| pectoral fin morphogenesis | 4 | 0.00381285982319801 |
| pectoral fin development | 9 | 0.00389726839216942 |

**Table S2. Significantly enriched GO terms at 2 dpf (bold terms were selected for Figure 4C) and 2.5 dpf (bold terms were selected for Figure 4D) compared between mutants and their siblings.**

| **GO_Term** | **S gene number** | ***p*-Value** |
| --- | --- | --- |
| **2 dpf** |  |  |
| lens development in camera-type eye | 30 | 0 |
| structural constituent of eye lens | 27 | 1.30327887804168E-19 |
| extracellular region | 91 | 4.31321645066873E-13 |
| extracellular space | 92 | 1.69144476203087E-10 |
| visual perception | 31 | 2.01688554746227E-10 |
| hemoglobin complex | 9 | 7.19050736711504E-09 |
| oxygen transport | 9 | 5.75535023389302E-07 |
| oxygen transporter activity | 9 | 5.75535023389302E-07 |
| oxygen binding | 9 | 5.75535023389302E-07 |
| immune response | 30 | 2.69394564555991E-06 |
| organic acid binding | 6 | 2.89811528473596E-06 |
| haptoglobin binding | 6 | 2.89811528473596E-06 |
| haptoglobin-hemoglobin complex | 6 | 2.89811528473596E-06 |
| hormone activity | 19 | 0.0000102988021603023 |
| calcium ion binding | 74 | 0.0000234164278086757 |
| positive regulation of heart contraction | 4 | 0.0000240001571923579 |
| hydrogen peroxide catabolic process | 7 | 0.0000531591349060001 |
| neuropeptide hormone activity | 9 | 0.0000584206184176539 |
| regulation of transcription, DNA-templated | 107 | 0.000103720247476291 |
| adult feeding behavior | 4 | 0.000113293251829631 |
| eye development | 13 | 0.000214871230243441 |
| cell adhesion | 37 | 0.000296226644231457 |
| cytokine activity | 15 | 0.000515167040228248 |
| **metal ion binding** | **242** | **0.000649359449140685** |
| negative regulation of appetite | 4 | 0.000707179557329511 |
| DNA binding | 131 | 0.000850153708132684 |
| hindbrain development | 10 | 0.000916446247774649 |
| peroxidase activity | 7 | 0.000978677661557126 |
| positive regulation of axon regeneration | 3 | 0.001301026703073 |
| basement membrane organization | 3 | 0.001301026703073 |
| retina vasculature development in camera-type eye | 3 | 0.001301026703073 |
| oocyte development | 3 | 0.001301026703073 |
| adenohypophysis development | 5 | 0.0013400972847567 |
| multicellular organismal development | 57 | 0.00144810856223254 |
| positive regulation of neurogenesis | 5 | 0.00196505118267964 |
| ectodermal placode development | 4 | 0.0022715139094015 |
| collagen trimer | 11 | 0.00272180833233793 |
| co-receptor binding | 3 | 0.00308262687993805 |
| epithalamus development | 3 | 0.00308262687993805 |
| ventricular cardiac muscle cell development | 3 | 0.00308262687993805 |
| RNA polymerase II core promoter proximal region sequence-specific DNA binding | 22 | 0.00336312543968598 |
| nucleic acid binding | 132 | 0.00348101671696843 |
| type B pancreatic cell differentiation | 4 | 0.00357687369063586 |
| macrophage differentiation | 4 | 0.00357687369063586 |
| iron ion binding | 21 | 0.00371290200637531 |
| fibroblast growth factor receptor signaling pathway | 7 | 0.00397900157947084 |
| cell chemotaxis | 7 | 0.00483409651106359 |
| apelin receptor binding | 2 | 0.0049058957337989 |
| regulation of myotome development | 2 | 0.0049058957337989 |
| T cell activation involved in immune response | 2 | 0.0049058957337989 |
| carboxyl- or carbamoyltransferase activity | 2 | 0.0049058957337989 |
| citrulline biosynthetic process | 2 | 0.0049058957337989 |
| RSF complex | 2 | 0.0049058957337989 |
| chromatin silencing at rDNA | 2 | 0.0049058957337989 |
| pancreatic epsilon cell differentiation | 2 | 0.0049058957337989 |
| dendritic cell antigen processing and presentation | 2 | 0.0049058957337989 |
| mRNA binding involved in posttranscriptional gene silencing | 2 | 0.0049058957337989 |
| detection of temperature stimulus involved in thermoception | 2 | 0.0049058957337989 |
| ventricular cardiac muscle cell action potential | 2 | 0.0049058957337989 |
| testosterone 6-beta-hydroxylase activity | 2 | 0.0049058957337989 |
| single-stranded DNA 5'-3' exodeoxyribonuclease activity | 2 | 0.0049058957337989 |
| rhombomere 4 development | 2 | 0.0049058957337989 |
| activation of MAPKK activity | 5 | 0.00509124957432405 |
| response to virus | 6 | 0.0052628789976209 |
| camera-type eye morphogenesis | 4 | 0.0053113389785906 |
| **cell dedifferentiation** | **3** | **0.00584432579971872** |
| palmitoyl hydrolase activity | 3 | 0.00584432579971872 |
| C-4 methylsterol oxidase activity | 3 | 0.00584432579971872 |
| receptor antagonist activity | 3 | 0.00584432579971872 |
| extracellular matrix | 20 | 0.00592254224242361 |
| positive regulation of cytosolic calcium ion concentration involved in phospholipase C-activating G-protein coupled signaling pathway | 6 | 0.0065213008144549 |
| cytokine binding | 6 | 0.0065213008144549 |
| response to lipopolysaccharide | 5 | 0.00664833893173356 |
| cellular response to heat | 5 | 0.00664833893173356 |
| fibroblast growth factor receptor binding | 5 | 0.00664833893173356 |
| calcium-mediated signaling | 8 | 0.00680407028533314 |
| embryonic hemopoiesis | 7 | 0.00694954807569026 |
| cardiac muscle cell proliferation | 4 | 0.00752953543132251 |
| cytokine receptor activity | 8 | 0.00790058403772542 |
| defense response to bacterium | 8 | 0.00790058403772542 |
| chemokine activity | 8 | 0.00790058403772542 |
| regulation of hematopoietic stem cell differentiation | 5 | 0.00851043611446978 |
| neutrophil differentiation | 5 | 0.00851043611446978 |
| positive regulation of cytosolic calcium ion concentration | 10 | 0.0093811068069779 |
| C-C chemokine binding | 6 | 0.00966947586652844 |
| chemokine binding | 6 | 0.00966947586652844 |
| protein homotrimerization | 3 | 0.00969718548151055 |
| ligand-gated ion channel activity | 3 | 0.00969718548151055 |
| response to X-ray | 3 | 0.00969718548151055 |
| piRNA metabolic process | 3 | 0.00969718548151055 |
| Cdc42 protein signal transduction | 3 | 0.00969718548151055 |
| response to exogenous dsRNA | 3 | 0.00969718548151055 |
| sequence-specific DNA binding transcription factor activity | 65 | 0.0101266191374031 |
| cellular response to starvation | 4 | 0.0102803968297963 |
| heme binding | 18 | 0.0104227590292092 |
| collagen fibril organization | 5 | 0.0107042879455717 |
| carboxypeptidase activity | 5 | 0.0107042879455717 |
| cellular iron ion homeostasis | 6 | 0.0115929978938281 |
| C-C chemokine receptor activity | 6 | 0.0115929978938281 |
| lymphangiogenesis | 7 | 0.0131279392932965 |
| heterotrimeric G-protein complex | 7 | 0.0131279392932965 |
| sterol biosynthetic process | 5 | 0.013254980907933 |
| cell projection assembly | 5 | 0.013254980907933 |
| misfolded protein binding | 4 | 0.013606535088643 |
| ferric iron binding | 4 | 0.013606535088643 |
| iron ion transport | 4 | 0.013606535088643 |
| receptor guanylyl cyclase signaling pathway | 4 | 0.013606535088643 |
| positive regulation of Rho protein signal transduction | 6 | 0.0137711046320993 |
| positive regulation of tyrosine phosphorylation of STAT protein | 2 | 0.0140311736140717 |
| intrinsic apoptotic signaling pathway | 2 | 0.0140311736140717 |
| hepatic duct development | 2 | 0.0140311736140717 |
| monocyte differentiation | 2 | 0.0140311736140717 |
| negative regulation of systemic arterial blood pressure | 2 | 0.0140311736140717 |
| calcium-independent cell-cell adhesion via plasma membrane cell-adhesion molecules | 2 | 0.0140311736140717 |
| aromatase activity | 2 | 0.0140311736140717 |
| annealing helicase activity | 2 | 0.0140311736140717 |
| positive regulation of circadian sleep/wake cycle, sleep | 2 | 0.0140311736140717 |
| steroid dehydrogenase activity | 2 | 0.0140311736140717 |
| extraocular skeletal muscle development | 2 | 0.0140311736140717 |
| negative regulation of circadian rhythm | 2 | 0.0140311736140717 |
| positive regulation of myeloid cell differentiation | 2 | 0.0140311736140717 |
| cornea development in camera-type eye | 2 | 0.0140311736140717 |
| amacrine cell differentiation | 2 | 0.0140311736140717 |
| cell migration involved in mesendoderm migration | 2 | 0.0140311736140717 |
| positive regulation of skeletal muscle fiber development | 2 | 0.0140311736140717 |
| lysozyme activity | 2 | 0.0140311736140717 |
| optic cup morphogenesis involved in camera-type eye development | 2 | 0.0140311736140717 |
| endothelin receptor activity | 2 | 0.0140311736140717 |
| water channel activity | 3 | 0.0147139660059822 |
| response to metal ion | 3 | 0.0147139660059822 |
| intestinal epithelial structure maintenance | 3 | 0.0147139660059822 |
| semicircular canal development | 3 | 0.0147139660059822 |
| melanosome organization | 3 | 0.0147139660059822 |
| intracellular sequestering of iron ion | 3 | 0.0147139660059822 |
| myelin sheath | 3 | 0.0147139660059822 |
| skeletal muscle cell differentiation | 3 | 0.0147139660059822 |
| extracellular matrix structural constituent | 10 | 0.0160262402764776 |
| cardiac muscle contraction | 5 | 0.016185656218059 |
| negative regulation of chromatin silencing | 5 | 0.016185656218059 |
| neuropeptide signaling pathway | 11 | 0.0164307407817342 |
| chemotaxis | 9 | 0.0169094919942884 |
| **neural crest cell development** | **7** | **0.0173731723731784** |
| vasculogenesis | 8 | 0.017450946208893 |
| atrioventricular canal development | 4 | 0.0175438681598509 |
| sequence-specific DNA binding | 54 | 0.019248141406075 |
| substrate adhesion-dependent cell spreading | 5 | 0.0195172749831851 |
| negative regulation of DNA recombination | 5 | 0.0195172749831851 |
| nucleosome positioning | 5 | 0.0195172749831851 |
| tumor necrosis factor receptor binding | 5 | 0.0195172749831851 |
| otic placode formation | 5 | 0.0195172749831851 |
| external side of plasma membrane | 16 | 0.0209112747444101 |
| cell motility | 3 | 0.0209351920542723 |
| positive regulation of myoblast differentiation | 3 | 0.0209351920542723 |
| pyridoxal phosphate binding | 8 | 0.0219630798873397 |
| integrin complex | 6 | 0.0219818029286436 |
| cellular_component | 127 | 0.022557534784388 |
| sequence-specific DNA binding RNA polymerase II transcription factor activity | 32 | 0.0230164538565384 |
| protein dimerization activity | 21 | 0.0242460327427292 |
| G-protein coupled receptor binding | 7 | 0.0254231404304394 |
| retinal cone cell differentiation | 2 | 0.0267613205250703 |
| spermidine acetylation | 2 | 0.0267613205250703 |
| thiol oxidase activity | 2 | 0.0267613205250703 |
| histone dephosphorylation | 2 | 0.0267613205250703 |
| negative regulation of activation of membrane attack complex | 2 | 0.0267613205250703 |
| rhombomere 4 morphogenesis | 2 | 0.0267613205250703 |
| negative regulation of extrinsic apoptotic signaling pathway in absence of ligand | 2 | 0.0267613205250703 |
| cellular response to corticotropin-releasing hormone stimulus | 2 | 0.0267613205250703 |
| pharyngeal muscle development | 2 | 0.0267613205250703 |
| positive regulation vascular endothelial growth factor production | 2 | 0.0267613205250703 |
| regulation of T cell differentiation | 2 | 0.0267613205250703 |
| response to fatty acid | 2 | 0.0267613205250703 |
| positive regulation of feeding behavior | 2 | 0.0267613205250703 |
| spermidine binding | 2 | 0.0267613205250703 |
| ventricular cardiac myofibril assembly | 2 | 0.0267613205250703 |
| neuromast deposition | 2 | 0.0267613205250703 |
| cGMP biosynthetic process | 4 | 0.0273615230599429 |
| myeloid cell differentiation | 5 | 0.0274552145732505 |
| growth factor activity | 14 | 0.0282340195681503 |
| filopodium | 3 | 0.028374471352974 |
| cellular response to unfolded protein | 3 | 0.028374471352974 |
| hematopoietic stem cell proliferation | 3 | 0.028374471352974 |
| blood vessel endothelial cell migration | 3 | 0.028374471352974 |
| epidermis development | 3 | 0.028374471352974 |
| palmitoyl-(protein) hydrolase activity | 3 | 0.028374471352974 |
| embryonic camera-type eye morphogenesis | 3 | 0.028374471352974 |
| determination of digestive tract left/right asymmetry | 3 | 0.028374471352974 |
| negative regulation of canonical Wnt signaling pathway | 7 | 0.0320181145502084 |
| notochord development | 7 | 0.0320181145502084 |
| RNA polymerase II transcription factor binding | 4 | 0.0332795756680027 |
| thiolester hydrolase activity | 4 | 0.0332795756680027 |
| cell-cell signaling | 4 | 0.0332795756680027 |
| biological_process | 106 | 0.0360985488976455 |
| double-stranded DNA binding | 8 | 0.0367100867610988 |
| regulation of cell growth | 3 | 0.0370231459933675 |
| ferroxidase activity | 3 | 0.0370231459933675 |
| fructose metabolic process | 3 | 0.0370231459933675 |
| **response to zinc ion** | **3** | **0.0370231459933675** |
| detection of mechanical stimulus involved in sensory perception | 3 | 0.0370231459933675 |
| pyrimidine nucleotide-sugar transmembrane transporter activity | 3 | 0.0370231459933675 |
| positive regulation of cell-substrate adhesion | 3 | 0.0370231459933675 |
| complement activation, classical pathway | 3 | 0.0370231459933675 |
| ATPase activity, coupled | 3 | 0.0370231459933675 |
| protein binding involved in protein folding | 3 | 0.0370231459933675 |
| cGMP-mediated signaling | 3 | 0.0370231459933675 |
| chromosome condensation | 5 | 0.0371869324862105 |
| anatomical structure morphogenesis | 6 | 0.0373108564412923 |
| ferrous iron binding | 4 | 0.0398846795489446 |
| oligodendrocyte differentiation | 4 | 0.0398846795489446 |
| cardiac muscle tissue development | 4 | 0.0398846795489446 |
| microtubule cytoskeleton | 8 | 0.0403029774038877 |
| angiogenesis | 17 | 0.041780530533792 |
| pancreatic A cell differentiation | 2 | 0.0425471162884919 |
| blood vessel maturation | 2 | 0.0425471162884919 |
| NAADP-sensitive calcium-release channel activity | 2 | 0.0425471162884919 |
| **embryonic skeletal joint development** | **2** | **0.0425471162884919** |
| coronary vasculature development | 2 | 0.0425471162884919 |
| branchiomeric skeletal muscle development | 2 | 0.0425471162884919 |
| regulation of vascular endothelial growth factor signaling pathway | 2 | 0.0425471162884919 |
| glucocorticoid receptor binding | 2 | 0.0425471162884919 |
| peptidoglycan catabolic process | 2 | 0.0425471162884919 |
| prechordal plate formation | 2 | 0.0425471162884919 |
| **chondrocyte proliferation** | **2** | **0.0425471162884919** |
| response to arsenic-containing substance | 2 | 0.0425471162884919 |
| retinal binding | 2 | 0.0425471162884919 |
| peptide hormone receptor binding | 2 | 0.0425471162884919 |
| small molecule binding | 2 | 0.0425471162884919 |
| hormone receptor binding | 2 | 0.0425471162884919 |
| adenylate cyclase-inhibiting adrenergic receptor signaling pathway | 2 | 0.0425471162884919 |
| response to insulin | 2 | 0.0425471162884919 |
| oxidoreductase activity, acting on paired donors, with incorporation or reduction of molecular oxygen, reduced ascorbate as one donor, and incorporation of one atom of oxygen | 2 | 0.0425471162884919 |
| lipopolysaccharide binding | 2 | 0.0425471162884919 |
| ectodermal placode formation | 2 | 0.0425471162884919 |
| alpha2-adrenergic receptor activity | 2 | 0.0425471162884919 |
| hemidesmosome assembly | 2 | 0.0425471162884919 |
| response to nutrient levels | 2 | 0.0425471162884919 |
| diamine N-acetyltransferase activity | 2 | 0.0425471162884919 |
| cytokine-mediated signaling pathway | 5 | 0.0427508313106413 |
| cell migration involved in gastrulation | 9 | 0.0433356800031676 |
| **cell differentiation** | **34** | **0.0467593655372417** |
| hindbrain morphogenesis | 3 | 0.0468543495140491 |
| perikaryon | 3 | 0.0468543495140491 |
| blood coagulation | 6 | 0.0470251081185209 |
| lymph vessel development | 4 | 0.047179777449788 |
| peptidase inhibitor activity | 4 | 0.047179777449788 |
| posterior lateral line neuromast primordium migration | 4 | 0.047179777449788 |
| skeletal muscle tissue development | 8 | 0.0481722271097471 |
| **2.5 dpf** |  |  |
| regulation of transcription, DNA-templated | 115 | 7.7715611723761E-16 |
| sequence-specific DNA binding | 68 | 2.0820123403098E-11 |
| extracellular matrix | 31 | 2.96676017086384E-11 |
| extracellular region | 72 | 8.88137341448214E-11 |
| extracellular space | 75 | 2.45027587109092E-09 |
| DNA binding | 124 | 3.51509865659239E-09 |
| multicellular organismal development | 60 | 3.6524641089386E-09 |
| sequence-specific DNA binding transcription factor activity | 68 | 5.04929503764018E-08 |
| protein dimerization activity | 29 | 5.95854466878265E-08 |
| extracellular matrix organization | 19 | 1.24412818158781E-07 |
| **cell differentiation** | **40** | **1.39120332276477E-06** |
| response to X-ray | 5 | 4.88656713784671E-06 |
| regulation of neurogenesis | 11 | 5.25563945286134E-06 |
| collagen trimer | 13 | 8.92333647883881E-06 |
| extracellular matrix structural constituent | 14 | 9.70442185743714E-06 |
| RNA polymerase II regulatory region sequence-specific DNA binding | 24 | 0.0000122251968592835 |
| cell surface | 19 | 0.0000159726146686801 |
| hormone activity | 16 | 0.0000213121073071587 |
| calcium ion binding | 58 | 0.000021324544635104 |
| synapse | 24 | 0.0000404432851809355 |
| lymphangiogenesis | 9 | 0.0000480770302003775 |
| neurological system process | 11 | 0.0000505515876930707 |
| positive regulation of T cell proliferation | 5 | 0.0000519332373907222 |
| filopodium | 5 | 0.0000519332373907222 |
| extracellular ligand-gated ion channel activity | 11 | 0.0000602084126539726 |
| ionotropic glutamate receptor activity | 8 | 0.0000623272451036883 |
| ion channel activity | 26 | 0.0000770747144582806 |
| ion transmembrane transport | 13 | 0.000105226390894941 |
| **mesenchyme migration** | **3** | **0.000110275057399966** |
| macromolecular complex | 9 | 0.000113920769486908 |
| hyaluronic acid binding | 7 | 0.00012902015277283 |
| palmitoyl hydrolase activity | 4 | 0.000164363601902675 |
| positive regulation of neurogenesis | 5 | 0.000351146648130962 |
| negative regulation of transcription from RNA polymerase II promoter | 23 | 0.000393124069918871 |
| endodermal cell differentiation | 3 | 0.000425266162216895 |
| cell body | 3 | 0.000425266162216895 |
| collagen type IX trimer | 3 | 0.000425266162216895 |
| positive regulation of skeletal muscle fiber development | 3 | 0.000425266162216895 |
| positive regulation of blood vessel endothelial cell proliferation involved in sprouting angiogenesis | 3 | 0.000425266162216895 |
| cone photoresponse recovery | 3 | 0.000425266162216895 |
| postsynaptic membrane | 14 | 0.000491795792526895 |
| peripheral nervous system neuron axonogenesis | 5 | 0.000505975337366849 |
| insulin-like growth factor II binding | 4 | 0.000547554651837201 |
| insulin-like growth factor I binding | 4 | 0.000547554651837201 |
| regulation of insulin-like growth factor receptor signaling pathway | 4 | 0.000547554651837201 |
| skeletal muscle cell differentiation | 4 | 0.000547554651837201 |
| eye development | 10 | 0.000687400011515349 |
| **negative regulation of Notch signaling pathway** | **6** | **0.000769565293519903** |
| cell adhesion | 28 | 0.00081437632026804 |
| sequence-specific DNA binding RNA polymerase II transcription factor activity | 29 | 0.000855225167544327 |
| transmembrane signaling receptor activity | 20 | 0.000897178510259389 |
| RNA polymerase II transcription regulatory region sequence-specific DNA binding transcription factor activity involved in negative regulation of transcription | 9 | 0.000989118234018482 |
| myofibril assembly | 7 | 0.000989964807775912 |
| forebrain development | 7 | 0.000989964807775912 |
| coronary vasculature development | 3 | 0.00102509449475799 |
| epithalamus development | 3 | 0.00102509449475799 |
| regulation of membrane potential | 12 | 0.00121389467225197 |
| myosin binding | 4 | 0.00132741245536394 |
| palmitoyl-(protein) hydrolase activity | 4 | 0.00132741245536394 |
| nervous system development | 20 | 0.00139723395948443 |
| anterior/posterior pattern specification | 12 | 0.00147344924786641 |
| ion transport | 40 | 0.00165226644857341 |
| thiolester hydrolase activity | 5 | 0.00166917387776133 |
| GABA-A receptor complex | 5 | 0.00166917387776133 |
| GABA-A receptor activity | 5 | 0.00166917387776133 |
| neuropeptide hormone activity | 6 | 0.00182818996448908 |
| visual perception | 16 | 0.00189149350604279 |
| thioesterase binding | 3 | 0.00197695512176466 |
| negative regulation of sequence-specific DNA binding transcription factor activity | 3 | 0.00197695512176466 |
| RNA polymerase II core promoter proximal region sequence-specific DNA binding | 18 | 0.00206179902889236 |
| calcium-dependent phospholipid binding | 9 | 0.00212774677651062 |
| positive regulation of neuron differentiation | 5 | 0.00213846909362658 |
| DNA rewinding | 2 | 0.00230179767699648 |
| negative regulation of developmental growth | 2 | 0.00230179767699648 |
| macrophage colony-stimulating factor receptor activity | 2 | 0.00230179767699648 |
| positive regulation of release of sequestered calcium ion into cytosol | 2 | 0.00230179767699648 |
| regulation of striated muscle contraction | 2 | 0.00230179767699648 |
| collagen fibril organization | 5 | 0.00269704472471521 |
| phospholipase inhibitor activity | 3 | 0.00333639876166125 |
| positive regulation of cell migration involved in sprouting angiogenesis | 3 | 0.00333639876166125 |
| G-protein coupled receptor internalization | 3 | 0.00333639876166125 |
| positive regulation of myoblast fusion | 3 | 0.00333639876166125 |
| G-protein beta/gamma-subunit complex | 4 | 0.00358756570054863 |
| positive regulation of ERK1 and ERK2 cascade | 10 | 0.0036754774929717 |
| insulin-like growth factor binding | 5 | 0.00411869208139248 |
| oligodendrocyte development | 4 | 0.00470814401443276 |
| G-protein beta-subunit binding | 4 | 0.00470814401443276 |
| photoreceptor outer segment | 5 | 0.00500004123609565 |
| collagen catabolic process | 5 | 0.00500004123609565 |
| cellular response to calcium ion | 6 | 0.00510955809950564 |
| response to metal ion | 3 | 0.00514850967344527 |
| positive regulation of G1/S transition of mitotic cell cycle | 3 | 0.00514850967344527 |
| angioblast cell migration from lateral mesoderm to midline | 3 | 0.00514850967344527 |
| negative regulation of cAMP-mediated signaling | 3 | 0.00514850967344527 |
| synaptic transmission, glycinergic | 3 | 0.00514850967344527 |
| **skeletal system development** | **7** | **0.0052851852382465** |
| hindbrain development | 7 | 0.00598120858528228 |
| response to nicotine | 4 | 0.00604185378360433 |
| regulation of muscle contraction | 4 | 0.00604185378360433 |
| prostate gland development | 2 | 0.00668484291618951 |
| negative regulation of interleukin-8 secretion | 2 | 0.00668484291618951 |
| alpha-beta T cell differentiation | 2 | 0.00668484291618951 |
| intrinsic apoptotic signaling pathway | 2 | 0.00668484291618951 |
| toll-like receptor 7 signaling pathway | 2 | 0.00668484291618951 |
| leukocyte migration | 2 | 0.00668484291618951 |
| positive regulation of G-protein coupled receptor protein signaling pathway | 2 | 0.00668484291618951 |
| positive regulation of neutrophil apoptotic process | 2 | 0.00668484291618951 |
| outflow tract morphogenesis | 2 | 0.00668484291618951 |
| negative regulation of T-helper 2 cell differentiation | 2 | 0.00668484291618951 |
| positive regulation of interleukin-2 production | 2 | 0.00668484291618951 |
| positive regulation of myeloid cell differentiation | 2 | 0.00668484291618951 |
| neutrophil clearance | 2 | 0.00668484291618951 |
| myoblast migration involved in skeletal muscle regeneration | 2 | 0.00668484291618951 |
| negative regulation of phospholipase A2 activity | 2 | 0.00668484291618951 |
| regulation of interleukin-1 production | 2 | 0.00668484291618951 |
| positive regulation of prostaglandin biosynthetic process | 2 | 0.00668484291618951 |
| response to interleukin-1 | 2 | 0.00668484291618951 |
| cornea development in camera-type eye | 2 | 0.00668484291618951 |
| amacrine cell differentiation | 2 | 0.00668484291618951 |
| positive regulation of T-helper 1 cell differentiation | 2 | 0.00668484291618951 |
| annealing helicase activity | 2 | 0.00668484291618951 |
| cellular response to glucocorticoid stimulus | 2 | 0.00668484291618951 |
| extraocular skeletal muscle development | 2 | 0.00668484291618951 |
| anatomical structure morphogenesis | 6 | 0.00682276743409194 |
| tumor necrosis factor receptor binding | 5 | 0.00714863360195472 |
| N-methyl-D-aspartate selective glutamate receptor activity | 3 | 0.00744898718424947 |
| organ development | 3 | 0.00744898718424947 |
| cilium-dependent cell motility | 3 | 0.00744898718424947 |
| N-methyl-D-aspartate selective glutamate receptor complex | 3 | 0.00744898718424947 |
| ectodermal placode development | 3 | 0.00744898718424947 |
| vasculogenesis | 7 | 0.00757539314793665 |
| cytokine binding | 5 | 0.0084332172022803 |
| RNA polymerase II transcription regulatory region sequence-specific DNA binding transcription factor activity involved in positive regulation of transcription | 8 | 0.00930755005117778 |
| acetylcholine-gated channel complex | 4 | 0.00941187266817012 |
| acetylcholine receptor activity | 4 | 0.00941187266817012 |
| embryonic camera-type eye morphogenesis | 3 | 0.0102651378654414 |
| arachidonic acid secretion | 3 | 0.0102651378654414 |
| positive regulation of myoblast differentiation | 3 | 0.0102651378654414 |
| cellular response to retinoic acid | 3 | 0.0102651378654414 |
| phototransduction | 6 | 0.0114355778411177 |
| cGMP binding | 4 | 0.0114765923456126 |
| osteoclast differentiation | 2 | 0.0129444196701624 |
| pharyngeal muscle development | 2 | 0.0129444196701624 |
| replication fork protection | 2 | 0.0129444196701624 |
| basement membrane organization | 2 | 0.0129444196701624 |
| oocyte development | 2 | 0.0129444196701624 |
| negative regulation of DNA replication | 2 | 0.0129444196701624 |
| cellular response to vascular endothelial growth factor stimulus | 2 | 0.0129444196701624 |
| glutamine biosynthetic process | 2 | 0.0129444196701624 |
| glutamate-ammonia ligase activity | 2 | 0.0129444196701624 |
| steroid dehydrogenase activity | 2 | 0.0129444196701624 |
| negative stranded viral RNA replication | 2 | 0.0129444196701624 |
| rhombomere 4 morphogenesis | 2 | 0.0129444196701624 |
| keratinocyte differentiation | 2 | 0.0129444196701624 |
| positive regulation of vesicle fusion | 2 | 0.0129444196701624 |
| positive regulation of wound healing | 2 | 0.0129444196701624 |
| apelin receptor activity | 2 | 0.0129444196701624 |
| neuron projection guidance | 2 | 0.0129444196701624 |
| positive regulation of cell-substrate adhesion | 3 | 0.01361678489422 |
| macrophage differentiation | 3 | 0.01361678489422 |
| neuromuscular synaptic transmission | 4 | 0.0138111231586089 |
| protein binding | 22 | 0.0152068949988566 |
| **positive regulation of cell migration** | **7** | **0.0156552195268694** |
| acetylcholine-gated cation channel activity | 4 | 0.0164262004371724 |
| autophagic vacuole membrane | 4 | 0.0164262004371724 |
| immune system process | 7 | 0.0171734955136784 |
| semaphorin receptor binding | 5 | 0.0172776492021777 |
| chemorepellent activity | 5 | 0.0172776492021777 |
| neuronal cell body | 6 | 0.0179010185180474 |
| syntaxin binding | 6 | 0.0179010185180474 |
| alpha-amino-3-hydroxy-5-methyl-4-isoxazolepropionic acid selective glutamate receptor complex | 4 | 0.0193311199680151 |
| positive regulation of gene expression | 5 | 0.0195803417297054 |
| muscle organ development | 5 | 0.0195803417297054 |
| tissue development | 6 | 0.0198419159457094 |
| positive regulation of epidermal growth factor receptor signaling pathway | 2 | 0.020890636453765 |
| sensory organ development | 2 | 0.020890636453765 |
| regulation of nodal signaling pathway | 2 | 0.020890636453765 |
| forebrain neuron differentiation | 2 | 0.020890636453765 |
| ectodermal placode formation | 2 | 0.020890636453765 |
| negative regulation of exocytosis | 2 | 0.020890636453765 |
| granulocyte chemotaxis | 2 | 0.020890636453765 |
| prechordal plate formation | 2 | 0.020890636453765 |
| neuron development | 6 | 0.0219210220497729 |
| galanin receptor activity | 3 | 0.0219733685406969 |
| skeletal myofibril assembly | 3 | 0.0219733685406969 |
| dendrite self-avoidance | 3 | 0.0219733685406969 |
| regulation of growth | 3 | 0.0219733685406969 |
| MyD88-dependent toll-like receptor signaling pathway | 3 | 0.0219733685406969 |
| negative regulation of axon extension involved in axon guidance | 5 | 0.0220753044245675 |
| otic placode formation | 4 | 0.0225337363651985 |
| notochord development | 6 | 0.0241421374414422 |
| G-protein coupled receptor binding | 6 | 0.0241421374414422 |
| G-protein coupled peptide receptor activity | 6 | 0.0265088151976577 |
| protein binding involved in cell-cell adhesion | 3 | 0.026987678406192 |
| water transmembrane transporter activity | 3 | 0.026987678406192 |
| striated muscle cell development | 3 | 0.026987678406192 |
| nitrogen compound metabolic process | 3 | 0.026987678406192 |
| G-protein coupled photoreceptor activity | 5 | 0.0276620004784892 |
| protein-chromophore linkage | 5 | 0.0276620004784892 |
| troponin complex | 4 | 0.0298563581572087 |
| lamellipodium | 4 | 0.0298563581572087 |
| photoreceptor outer segment membrane | 2 | 0.030347477119765 |
| olfactory placode development | 2 | 0.030347477119765 |
| complement activation, lectin pathway | 2 | 0.030347477119765 |
| adult feeding behavior | 2 | 0.030347477119765 |
| cellular response to hydrogen peroxide | 2 | 0.030347477119765 |
| T cell costimulation | 2 | 0.030347477119765 |
| alpha-amino-3-hydroxy-5-methyl-4-isoxazole propionate selective glutamate receptor activity | 2 | 0.030347477119765 |
| phospholipase A2 inhibitor activity | 2 | 0.030347477119765 |
| photoreceptor activity | 5 | 0.0307623507347669 |
| steroid hormone receptor activity | 8 | 0.0310777322700269 |
| immune response | 19 | 0.0317215611268283 |
| macrophage chemotaxis | 3 | 0.0325575620041314 |
| response to cold | 3 | 0.0325575620041314 |
| long-term synaptic potentiation | 3 | 0.0325575620041314 |
| cortical actin cytoskeleton | 3 | 0.0325575620041314 |
| regulation of synaptic transmission | 4 | 0.0339850353490728 |
| cellular response to light stimulus | 5 | 0.0340721868747257 |
| nucleus | 203 | 0.0342676411789409 |
| homophilic cell adhesion via plasma membrane adhesion molecules | 13 | 0.0360526047325471 |
| cytokine activity | 9 | 0.0365096513396562 |
| perinuclear region of cytoplasm | 7 | 0.0383735494248534 |
| **Notch binding** | **3** | **0.038676571593484** |
| positive regulation of synaptic transmission, glutamatergic | 3 | 0.038676571593484 |
| neuropeptide signaling pathway | 8 | 0.0402933285206766 |
| regulation of hormone secretion | 2 | 0.0411519084630068 |
| regulation of leukocyte migration | 2 | 0.0411519084630068 |
| sarcomerogenesis | 2 | 0.0411519084630068 |
| extracellular-glycine-gated chloride channel activity | 2 | 0.0411519084630068 |
| intrinsic apoptotic signaling pathway in response to DNA damage by p53 class mediator | 2 | 0.0411519084630068 |
| dense core granule | 2 | 0.0411519084630068 |
| paraxial mesoderm development | 2 | 0.0411519084630068 |
| protein homotrimerization | 2 | 0.0411519084630068 |
| double-stranded RNA adenosine deaminase activity | 2 | 0.0411519084630068 |
| Cdc42 protein signal transduction | 2 | 0.0411519084630068 |
| nuclear replication fork | 2 | 0.0411519084630068 |
| positive regulation of protein autophosphorylation | 2 | 0.0411519084630068 |
| negative regulation of T cell proliferation | 2 | 0.0411519084630068 |
| piRNA metabolic process | 2 | 0.0411519084630068 |
| platelet-derived growth factor receptor binding | 2 | 0.0411519084630068 |
| phospholipid dephosphorylation | 3 | 0.0453348068346151 |
| myeloid cell development | 3 | 0.0453348068346151 |
| lipid phosphatase activity | 3 | 0.0453348068346151 |
| acetylcholine binding | 3 | 0.0453348068346151 |
| erythrocyte maturation | 3 | 0.0453348068346151 |
| PDZ domain binding | 3 | 0.0453348068346151 |
| cell migration involved in gastrulation | 7 | 0.0470857254455472 |
| substrate-dependent cell migration, cell extension | 1 | 0.048 |
| vascular smooth muscle cell development | 1 | 0.048 |
| positive regulation of macrophage chemotaxis | 1 | 0.048 |
| positive regulation of trophoblast cell migration | 1 | 0.048 |
| endothelial cell activation involved in immune response | 1 | 0.048 |
| lateral line ganglion development | 1 | 0.048 |
| type 3 melanocortin receptor binding | 1 | 0.048 |
| induction by virus of host apoptotic process | 1 | 0.048 |
| signal transduction by p53 class mediator | 1 | 0.048 |
| myoblast proliferation involved in skeletal muscle regeneration | 1 | 0.048 |
| kynurenine 3-monooxygenase activity | 1 | 0.048 |
| phospholipid homeostasis | 1 | 0.048 |
| negative regulation of transposition, RNA-mediated | 1 | 0.048 |
| regulation of toll-like receptor signaling pathway | 1 | 0.048 |
| response to osmotic stress | 1 | 0.048 |
| toll-like receptor 9 signaling pathway | 1 | 0.048 |
| pericardium morphogenesis | 1 | 0.048 |
| transmembrane transporter complex | 1 | 0.048 |
| CTP binding | 1 | 0.048 |
| osteoclast development | 1 | 0.048 |
| toll-like receptor 6 signaling pathway | 1 | 0.048 |
| choline metabolic process | 1 | 0.048 |
| negative regulation of cell division | 1 | 0.048 |
| cholesterol 25-hydroxylase activity | 1 | 0.048 |
| relaxation of skeletal muscle | 1 | 0.048 |
| positive regulation of neuron apoptotic process | 1 | 0.048 |
| glycerophosphocholine cholinephosphodiesterase activity | 1 | 0.048 |
| adult heart development | 1 | 0.048 |
| beta-N-acetylglucosaminidase activity | 1 | 0.048 |
| myoblast differentiation involved in skeletal muscle regeneration | 1 | 0.048 |
| ventral spinal cord interneuron fate commitment | 1 | 0.048 |
| dimethylglycine dehydrogenase activity | 1 | 0.048 |
| serine family amino acid catabolic process | 1 | 0.048 |
| endoplasmic reticulum polarization | 1 | 0.048 |
| low-density lipoprotein particle remodeling | 1 | 0.048 |
| RNA-directed DNA polymerase activity | 1 | 0.048 |
| actin filament bundle retrograde transport | 1 | 0.048 |
| protein-succinyllysine desuccinylase activity | 1 | 0.048 |
| regulation of cell aging | 1 | 0.048 |
| cytoplasmic actin-based contraction involved in cell motility | 1 | 0.048 |
| L-allo-threonine aldolase activity | 1 | 0.048 |
| mesendoderm migration | 1 | 0.048 |
| cellular response to alcohol | 1 | 0.048 |
| paraxial mesodermal cell fate specification | 1 | 0.048 |
| growth hormone secretion | 1 | 0.048 |
| age-dependent general metabolic decline | 1 | 0.048 |
| error-free translesion synthesis | 1 | 0.048 |
| centriole elongation | 1 | 0.048 |
| hindbrain maturation | 1 | 0.048 |
| neuroblast fate determination | 1 | 0.048 |
| interleukin-6 binding | 1 | 0.048 |
| telomerase catalytic core complex | 1 | 0.048 |
| glycine:sodium symporter activity | 1 | 0.048 |
| regulation of cellular response to insulin stimulus | 1 | 0.048 |
| threonine biosynthetic process | 1 | 0.048 |
| leukemia inhibitory factor receptor activity | 1 | 0.048 |
| thymidine catabolic process | 1 | 0.048 |
| positive regulation of growth hormone secretion | 1 | 0.048 |
| apelin receptor signaling pathway | 1 | 0.048 |
| positive regulation of hh target transcription factor activity | 1 | 0.048 |
| response to vitamin K | 1 | 0.048 |
| regulation of ketone biosynthetic process | 1 | 0.048 |
| triglyceride binding | 1 | 0.048 |
| beta-alanine metabolic process | 1 | 0.048 |
| positive regulation of multicellular organism growth | 1 | 0.048 |
| neuromast hair cell differentiation | 1 | 0.048 |
| growth hormone-releasing hormone receptor binding | 1 | 0.048 |
| L-fucose mutarotase activity | 1 | 0.048 |
| regulation of systemic arterial blood pressure | 1 | 0.048 |
| structural constituent of bone | 1 | 0.048 |
| interleukin-6 receptor activity | 1 | 0.048 |
| acetylcholine receptor inhibitor activity | 1 | 0.048 |
| protein-DNA complex | 1 | 0.048 |
| regulation of macrophage differentiation | 1 | 0.048 |
| positive regulation of granuloma formation | 1 | 0.048 |
| regulation of glucose import | 1 | 0.048 |
| nitric-oxide synthase regulator activity | 1 | 0.048 |
| heterocyclic compound binding | 1 | 0.048 |
| mitotic G1/S transition checkpoint | 1 | 0.048 |
| glycine biosynthetic process | 1 | 0.048 |
| leukotriene B4 receptor activity | 1 | 0.048 |
| insulin-like growth factor binding protein complex | 1 | 0.048 |
| detection of diacyl bacterial lipopeptide | 1 | 0.048 |
| regulation of synapse maturation | 1 | 0.048 |
| telencephalon regionalization | 1 | 0.048 |
| lactose binding | 1 | 0.048 |
| interleukin-6 receptor complex | 1 | 0.048 |
| voltage-gated potassium channel activity involved in ventricular cardiac muscle cell action potential repolarization | 1 | 0.048 |
| peptidyl-cysteine oxidation | 1 | 0.048 |
| granulosa cell development | 1 | 0.048 |
| membrane repolarization during action potential | 1 | 0.048 |
| glycoprotein metabolic process | 1 | 0.048 |
| oncostatin-M receptor complex | 1 | 0.048 |
| 2-oxobutyrate biosynthetic process | 1 | 0.048 |
| glial cell fate determination | 1 | 0.048 |
| karyomere membrane fusion | 1 | 0.048 |
| beta-ureidopropionase activity | 1 | 0.048 |
| hydroxyapatite binding | 1 | 0.048 |
| amino-acid betaine transport | 1 | 0.048 |
| protein-malonyllysine demalonylase activity | 1 | 0.048 |
| regulation of T cell migration | 1 | 0.048 |
| beta2-adrenergic receptor activity | 1 | 0.048 |
| beta-alanine biosynthetic process via 3-ureidopropionate | 1 | 0.048 |
| negative regulation of oocyte development | 1 | 0.048 |
| positive regulation of fast-twitch skeletal muscle fiber contraction | 1 | 0.048 |
| left-handed Z-DNA binding | 1 | 0.048 |
| H zone | 1 | 0.048 |
| amino-acid betaine transmembrane transporter activity | 1 | 0.048 |
| negative regulation of axial mesodermal cell fate specification | 1 | 0.048 |
| negative regulation of male gonad development | 1 | 0.048 |
| DNA strand elongation | 1 | 0.048 |
| DNA damage response, signal transduction by p53 class mediator resulting in transcription of p21 class mediator | 1 | 0.048 |
| negative regulation of delayed rectifier potassium channel activity | 1 | 0.048 |
| oocyte differentiation | 1 | 0.048 |
| telomeric template RNA reverse transcriptase activity | 1 | 0.048 |
| compound eye photoreceptor cell differentiation | 1 | 0.048 |
| negative regulation of striated muscle contraction | 1 | 0.048 |
| regulation of myofibril size | 1 | 0.048 |
| invagination involved in gastrulation with mouth forming second | 1 | 0.048 |
| monosaccharide metabolic process | 1 | 0.048 |
| B cell chemotaxis | 1 | 0.048 |
| cortical microtubule organization | 1 | 0.048 |
| muscle tendon junction | 1 | 0.048 |
| immune response in brain or nervous system | 1 | 0.048 |
| threonine synthase activity | 1 | 0.048 |
| 2-hydroxyglutarate dehydrogenase activity | 1 | 0.048 |
| triglyceride transport | 1 | 0.048 |
| karyogamy | 1 | 0.048 |
| ciliary neurotrophic factor receptor complex | 1 | 0.048 |
| vitelline membrane formation | 1 | 0.048 |
| negative regulation of multicellular organism growth | 1 | 0.048 |
| negative regulation of ryanodine-sensitive calcium-release channel activity | 1 | 0.048 |
| negative regulation of muscle contraction | 1 | 0.048 |
| heterotrimeric G-protein complex | 5 | 0.04945617942109 |
| integral component of plasma membrane | 58 | 0.049579451240539 |

**Table S3. Fold change in domain-specific genes compared between mutants and their siblings at 2 dpf and 2.5 dpf.**

| **Gene name** | **log_2_(fc)** | **Domain** |
| --- | --- | --- |
| **2 dpf** |  |  |
| *nr2f1a* | 0.102180710010732 | D |
| *nr2f1b* | -0.329803226788816 | D |
| *nr2f2* | 0.00956773833729945 | D |
| *nr2f5* | 0.827375865134156 | D |
| *nr2f6a* | 0.398224577255092 | D |
| *nr2f6b* | 0.931453788645687 | D |
| *jag1b* | 1.52683768134575 | D |
| *hey1* | 0.0000090230228147126 | D |
| *ednraa* | 1.04606109215638 | D |
| *ednrab* | 1.3898654545184 | D |
| *pou3f3b* | 0.357953951086691 | D |
| *pou3f3a* | 0.344646926666663 | D |
| *grem2b* | 0.706347603799926 | D |
| *prss35* | 1.42746865629834 | D |
| *fmoda* | 0.855410850470404 | D |
| *kera* | 0.580366249315259 | D |
| *emp2* | 0.326308653768866 | D |
| *CALHM2 (1 of many)* | 1.73343569641887 | D |
| *cdh11* | 0.637618533456761 | D |
| *serpinf1* | 0.60379045571658 | D |
| *emilin1a* | 0.081218508467835 | D |
| *postnb* | 1.2882672234951 | D |
| *cd248a* | 0.599710581415002 | D |
| *arl4ca* | 0.4522579664415 | D |
| *zfp36l1b* | 0.149551207834574 | D |
| *thbs1b* | 1.07046160270293 | D |
| *tril* | 0.532108483843735 | D |
| *cxcl12b* | 0.57888633700051 | D |
| *dse* | 0.0709505379549268 | D |
| *mn1a* | 0.31671968650645 | D |
| *bmp2b* | 1.43657504373041 | D |
| *kctd15a* | -0.0549137846237852 | D |
| *snai1a* | 0.8650660604902 | D |
| *tfap2a* | 0.325787000647341 | D |
| *fgf20b* | 2.56396861886931 | D |
| *twist1a* | 0.638969417136983 | D |
| *mef2ca* | 0.163712942198918 | I |
| *mef2cb* | -0.0462018999926589 | I |
| *nkx3.2* | -1.96301899891515 | I |
| *emx2* | 0.805775627046715 | I |
| *irx7* | -1.69037091543628 | I |
| *zgc:162612* | 0.55991709138333 | I |
| *igfbp5b* | 0.613921864329186 | I |
| *ctgfb* | -1.06727559477283 | I |
| *ms4a17a.11* | 1.00548789543402 | I |
| *foxd1* | 0.653982317376532 | I |
| *spon2b* | -1.24320950010457 | I |
| *rgmd* | 0.653968016165678 | I |
| *her6* | 0.147564902606063 | I |
| *hand2* | 0.678179059825948 | V |
| *sema3bl* | -0.111396813685212 | V |
| *fzd9b* | -0.852213870474559 | V |
| *foxf2a* | -0.763769866587376 | V |
| *dcps* | 0.0617230108559777 | V |
| *smad6a* | 0.959169370420856 | V |
| *cep57l1* | 0.61523682987266 | V |
| *foxf1* | 0.0319790774746585 | V |
| *skp2* | 0.987235460972822 | V |
| *crabp2b* | -0.566790002315344 | V |
| *pitx1* | 1.12925746387897 | V |
| *twist1b* | 0.623029198883438 | V |
| *lrrn3a* | 0.0388919463961172 | V |
| *sumo3b* | 0.408146306216456 | V |
| *barx1* | -0.791906648962588 | V |
| *mrrf* | 0.58853267317344 | V |
| *satb2* | 0.289850320573877 | V |
| *tmem107l* | 0.224229872217346 | V |
| *epha4b* | 0.404816710432459 | VI |
| *dlx1a* | 0.419439002743257 | VI |
| *dlx2a* | 0.389026173321672 | VI |
| *dlx2b* | 0.173903953674687 | VI |
| *dlx3b* | -0.306241119510378 | VI |
| *dlx4b* | -0.435753614397584 | VI |
| *dlx5a* | -0.153624587854664 | VI |
| *dlx6a* | 0.808148352500081 | VI |
| *msx1a* | 0.690527463004461 | VI |
| *msx1b* | 0.857390021599445 | VI |
| *msx2a* | 0.53214643596205 | VI |
| *msx2b* | 1.16683444090404 | VI |
| *fgfbp2a* | -0.510647795535403 | VI |
| *si:dkey-16p21.8* | 0.589648244795939 | VI |
| *shox* | -0.10726692075017 | VI |
| *gsc* | 0.0443595830182959 | VI |
| *stmn1a* | 0.53054891099201 | VI |
| *si:ch211-282k23.2* | 1.61641882605444 | VI |
| *si:ch211-222l21.1* | -0.116506414706925 | VI |
| *otud4* | 0.870286553807765 | VI |
| *ift22* | 0.207316715692843 | VI |
| *id3* | -0.271628506842006 | VI |
| *tmem119b* | 0.0215627324195384 | VI |
| *AL929378.1* | 0.407009335256789 | VI |
| *dlx4a* | -1.51953350315904 | VI |
| **2.5 dpf** |  |  |
| *nr2f5* | 0.804119218761807 | D |
| *nr2f6b* | 0.337250142238356 | D |
| *nr2f1b* | -0.269764858055136 | D |
| *nr2f1a* | -0.143275992998519 | D |
| *nr2f6a* | -0.06181204815866 | D |
| *nr2f2* | -0.0223859634376406 | D |
| *jag1b* | 1.28027471637736 | D |
| *hey1* | -0.273714966454807 | D |
| *ednraa* | 0.677666604123756 | D |
| *ednrab* | 0.229539569038459 | D |
| *pou3f3a* | -0.0578498398772247 | D |
| *pou3f3b* | -0.173012025224358 | D |
| *grem2b* | 0.0442962751737519 | D |
| *prss35* | 0.315892070112209 | D |
| *fmoda* | 0.728740077442362 | D |
| *kera* | 0.0133025275603519 | D |
| *emp2* | 0.48302805554332 | D |
| *CALHM2 (1 of many)* | 1.94435420792878 | D |
| *cdh11* | 0.363770024465535 | D |
| *serpinf1* | 0.0356724584841675 | D |
| *emilin1a* | -0.0588929880020332 | D |
| *postnb* | 1.09406119956484 | D |
| *cd248a* | 0.353927543644285 | D |
| *arl4ca* | -0.12088914339976 | D |
| *zfp36l1b* | -0.118496060371728 | D |
| *thbs1b* | 0.252110528344955 | D |
| *tril* | -0.0854454502740764 | D |
| *cxcl12b* | -0.0224946852562299 | D |
| *dse* | 0.294729651527007 | D |
| *mn1a* | 0.264067505516739 | D |
| *bmp2b* | 0.866437791287509 | D |
| *kctd15a* | -0.00538861281819895 | D |
| *snai1a* | 0.578612277196243 | D |
| *tfap2a* | -0.394886935419582 | D |
| *fgf20b* | 0.79091299740911 | D |
| *twist1a* | 0.832782367865777 | D |
| *mef2ca* | -0.334048187795157 | I |
| *mef2cb* | -0.615142893973956 | I |
| *nkx3.2* | -1.38488189598042 | I |
| *emx2* | 0.216823248257924 | I |
| *irx7* | -1.51167877447042 | I |
| *fsta* | 1.22956374920405 | I |
| *zgc:162612* | 1.07829057237595 | I |
| *igfbp5b* | 0.441496007246175 | I |
| *ctgfb* | -1.13251345759688 | I |
| *ms4a17a.11* | 1.08636004107837 | I |
| *foxd1* | 0.737946260193854 | I |
| *spon2b* | -0.821603678581942 | I |
| *rgmd* | 0.374948753644054 | I |
| *her6* | 0.0490560072168477 | I |
| *hand2* | 0.117614313697615 | V |
| *sema3bl* | -1.0784448356531 | V |
| *fzd9b* | -1.71808883033382 | V |
| *foxf2a* | -0.608628034072852 | V |
| *dcps* | 0.120811506462543 | V |
| *smad6a* | 0.47192577892704 | V |
| *cep57l1* | 0.195655668200035 | V |
| *foxf1* | 0.102834833539669 | V |
| *skp2* | 0.564871990604765 | V |
| *crabp2b* | -0.625928709606732 | V |
| *pitx1* | 0.670868064577094 | V |
| *twist1b* | 0.71374561821475 | V |
| *lrrn3a* | -0.703803623282985 | V |
| *sumo3b* | 0.381932082969608 | V |
| *barx1* | -0.348798811860472 | V |
| *mrrf* | 0.777710864698941 | V |
| *satb2* | 0.210064198967743 | V |
| *tmem107l* | 0.126003472220709 | V |
| *dlx1a* | 0.111932292266019 | VI |
| *dlx2a* | 0.196417982432902 | VI |
| *dlx2b* | 0.778241016167643 | VI |
| *dlx3b* | -0.323431897634591 | VI |
| *dlx4b* | -0.25109574347032 | VI |
| *dlx5a* | -0.213180149514423 | VI |
| *dlx6a* | 0.524121019664098 | VI |
| *msx1a* | 0.6404544844458 | VI |
| *msx1b* | 0.903785791211983 | VI |
| *msx2b* | 0.700957197135962 | VI |
| *epha4b* | -0.110315915413007 | VI |
| *fgfbp2a* | -0.936208053312719 | VI |
| *si:dkey-16p21.8* | -0.312306959087256 | VI |
| *shox* | -0.391380667695193 | VI |
| *gsc* | 0.730173879618186 | VI |
| *stmn1a* | 0.548556406974909 | VI |
| *si:ch211-282k23.2* | -0.512611656496472 | VI |
| *si:ch211-222l21.1* | 0.0782025668428289 | VI |
| *otud4* | -0.978661567919945 | VI |
| *ift22* | 0.132079214026215 | VI |
| *id3* | -0.0699777806737994 | VI |
| *tmem119b* | 0.260064142460488 | VI |
| *dlx4a* | -1.51833020829847 | VI |

**Table S4. Canonical markers along the AP and DV axes at 2 dpf and 2.5 dpf used for the volcano plots.**

| **Gene_name** | **log_2_(fc)** | ***p*-Value** |
| --- | --- | --- |
| **2 dpf** |  |  |
| *nr2f1a* | 0.102180710010732 | 0.543118864355161 |
| *nr2f1b* | -0.329803226788816 | 0.0437715071102868 |
| *nr2f2* | 0.00956773833729945 | 0.393111092787881 |
| *nr2f5* | 0.827375865134156 | 0.0370916259159999Table |
| *nr2f6a* | 0.398224577255092 | 0.552844906383558 |
| *nr2f6b* | 0.931453788645687 | 0.0118728707598881 |
| *jag1b* | 1.52683768134575 | 0.000114183491461592 |
| *hey1* | 0.0000090230228147126 | 0.403253023524825 |
| *dlx1a* | 0.419439002743257 | 0.504668375066131 |
| *dlx2a* | 0.389026173321672 | 0.561819729583236 |
| *dlx2b* | 0.173903953674687 | 0.862835833459743 |
| *dlx3b* | -0.306241119510378 | 0.0609241686289093 |
| *dlx4a* | -1.51953350315904 | 5.27589968280561E-07 |
| *dlx4b* | -0.435753614397584 | 0.0570161173346557 |
| *dlx5a* | -0.153624587854664 | 0.15607665301223 |
| *dlx6a* | 0.808148352500081 | 0.0820050491808487 |
| *msx1a* | 0.690527463004461 | 0.206974733658006 |
| *msx1b* | 0.857390021599445 | 0.0147596248252879 |
| *msx2a* | 0.53214643596205 | 1 |
| *msx2b* | 1.16683444090404 | 0.00090263743453852 |
| *hand2* | 0.678179059825948 | 0.11600116989511 |
| *ednraa* | 1.04606109215638 | 0.00883781440697478 |
| *ednrab* | 1.3898654545184 | 0.0069484831458423 |
| *mef2ca* | 0.163712942198918 | 0.86518461196693 |
| *mef2cb* | -0.0462018999926589 | 0.32890405237266 |
| *nkx3.2* | -1.96301899891515 | 0.000000000107613827969318 |
| *epha4b* | 0.404816710432459 | 0.59392827302696 |
| *pou3f3b* | 0.357953951086691 | 0.692033310526873 |
| *pou3f3a* | 0.344646926666663 | 0.739837303408061 |
| *grem2b* | 0.706347603799926 | 0.174219423654195 |
| *hoxb1a* | 1.49780740384558 | 0.0122091331314815 |
| *hoxb1b* | 1.24141937845169 | 0.0184335615198565 |
| *hoxa2b* | -0.111361277115372 | 0.256068086342249 |
| *hoxb2a* | 0.262483968598138 | 0.967206650722546 |
| *hoxa3a* | -0.0479158708664624 | 0.240066214598999 |
| *hoxb3a* | -0.1965649846394 | 0.0685402363048944 |
| *hoxd3a* | 0.455126063440767 | 0.553549986718678 |
| *hoxb4a* | -0.403048473984456 | 0.0455176421090196 |
| *hoxd4a* | 0.0153118841812043 | 0.416802306969999 |
| *hoxa1a* | 0.604758606313559 | 0.479964211495575 |
| **2.5 dpf** |  |  |
| *nr2f5* | 0.804119218761807 | 0.0245340519010492 |
| *nr2f6b* | 0.337250142238356 | 0.290548274166152 |
| *nr2f1b* | -0.269764858055136 | 0.367727492571362 |
| *nr2f1a* | -0.143275992998519 | 0.5815546107737 |
| *nr2f6a* | -0.06181204815866 | 0.80173820123561 |
| *nr2f2* | -0.0223859634376406 | 0.889127264694332 |
| *jag1b* | 1.28027471637736 | 0.000317608141595076 |
| *hey1* | -0.273714966454807 | 0.465558727145216 |
| *dlx1a* | 0.111932292266019 | 0.760419173441214 |
| *dlx2a* | 0.196417982432902 | 0.553883913360936 |
| *dlx2b* | 0.778241016167643 | 0.136578189894094 |
| *dlx3b* | -0.323431897634591 | 0.336971638737194 |
| *dlx4a* | -1.51833020829847 | 2.29898149413686E-06 |
| *dlx4b* | -0.25109574347032 | 0.419830929916253 |
| *dlx5a* | -0.213180149514423 | 0.421600102248434 |
| *dlx6a* | 0.524121019664098 | 0.127661761660276 |
| *msx1a* | 0.6404544844458 | 0.0796217101796634 |
| *msx1b* | 0.903785791211983 | 0.0440644854937872 |
| *msx2a* | 2.67606899996639 | 1 |
| *msx2b* | 0.700957197135962 | 0.0829407388393673 |
| *hand2* | 0.117614313697615 | 0.773568068713372 |
| *ednraa* | 0.677666604123756 | 0.0332051730450111 |
| *ednrab* | 0.229539569038459 | 0.710346587590173 |
| *mef2ca* | -0.334048187795157 | 0.479864159020706 |
| *mef2cb* | -0.615142893973956 | 0.0627959712624308 |
| *nkx3.2* | -1.38488189598042 | 0.000429640620568771 |
| *epha4b* | -0.110315915413007 | 0.673125670939346 |
| *pou3f3a* | -0.0578498398772247 | 0.807045539735278 |
| *pou3f3b* | -0.173012025224358 | 0.544592052652718 |
| *grem2b* | 0.0442962751737519 | 0.971764761565651 |
| *hoxb1a* | 1.07863566631592 | 0.0415641728294486 |
| *hoxb1b* | 0.669260601044376 | 0.158136439682158 |
| *hoxa2b* | 0.492672859776278 | 0.133932321580436 |
| *hoxb2a* | 0.149299567381973 | 0.721015121309073 |
| *hoxa3a* | -0.0363607628315283 | 0.83761968705633 |
| *hoxb3a* | -0.396483033871922 | 0.175265754347929 |
| *hoxd3a* | -0.0385775432191548 | 0.831552618370819 |
| *hoxb4a* | -0.625484050807516 | 0.0847605899164927 |
| *hoxd4a* | -0.285382917129169 | 0.406740414056266 |
| *hoxa1a* | 0.597284813145374 | 0.330287955371717 |

**Table S5. The morpholino sequences used in this study.**

| **Gene** | **Sequence** | **Reference** |
| --- | --- | --- |
| *jag1b* | CTGAACTCCGTCGCAGAATCATGCC | PMID: 28974684 |
| *notch2* | AGGTGAACACTTACTTCATGCCAAA | PMID: 22368273 |

**Table S6. Primer pairs used for ChIP-qPCR.**

| **Region** | **Sequence 5’-3’** |
| --- | --- |
| P1 | Forward: TGATCATTGGGTGGAGGAGGACTG |
|  | Reverse: CCATAATTGGGAAGGGCATATCCC |
| P2 | Forward: CGGGAATGGTTAGAATGAGATGCA |
|  | Reverse: CCCAGCATAGATAGTGCCCCATAC |
| P3 | Forward: ACGACAGCGGTTAGGTTCTTTTCA |
|  | Reverse: GACTTCGGGAAGGGCTTTCTGAGA |
| P4 | Forward: TACACCTGGGGCTTGCAAGG |
|  | Reverse: AGGAGAAAGGAACCTGGAAGGAC |
| P-*Cdh1* | Forward: AGTTCCTTGGCTGCCACCT |
|  | Reverse: ACCAGTGAGCAGCGCAGA |
